# Supplementary material for: Cytochrome P450 Can Epoxidize an Oxepin to a Reactive 2,3-Epoxyoxepin Intermediate: Potential Insights into Metabolic Ring-Opening of Benzene
Source: Molecules. 2020 Oct 3;25(19):4542. doi: 10.3390/molecules25194542 (PMC7582548; doi:10.3390/molecules25194542)
Supplement: Supplementary file 1 [file molecules-25-04542-s001.pdf]

Supporting Information

Article

Cytochrome P450 Can Epoxidize an Oxepin to a Reactive 2,3-Epoxyoxepin Intermediate: Potential Insights into Metabolic Ring-Opening of Benzene

Holly M. Weaver-Guevara [1,*], Ryan W. Fitzgerald [2], Noah A. Cote [2] and Arthur Greenberg [2,*]

[1] Department of Chemistry, Grove City College, Grove City, PA 16127, USA
[2] Department of Chemistry, University of New Hampshire, Durham, NH 03824, USA; rwj9@wildcats.unh.edu (R.W.F.); nac1009@wildcats.unh.edu (N.A.C.)
[*] Correspondence: guevarahm@gcc.edu (H.M.W.-G.); art.greenberg@unh.edu (A.G.); Tel.: +1-603-862-1180 (A.G.)

Academic Editor: Edward Lee-Ruff
Received: 10 September 2020; Accepted: 1 October 2020; Published: 3 October 2020

Table of Contents:

1. Alternative synthesis of 4,5-benzoxepin S2

2. Characterization S4

2.1. Enzymatic Incubations with **11** S4

2.2. Enzymatic Incubations with **8** S8

2.3. Synthesis of 4,5-benzoxepin—NMR S11

2.4. Characterization of **20** S15

2.5. Characterization of **21** S24

3. Calculations S35

4. References S36

1. Alternative Synthesis of 4,5-benzoxepin

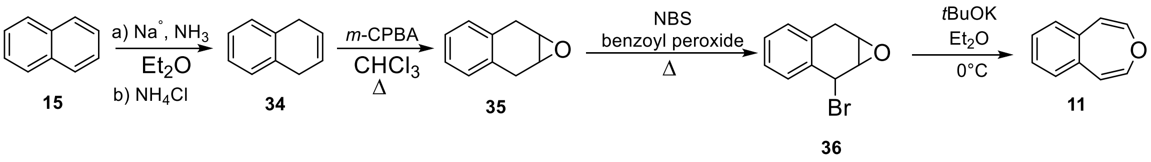

Scheme S1. Synthetic route to 4,5-benzoxepin.

*Preparation of 1,4-dihydronaphthalene (**34**).*

An oven-dried, 500 mL 3-necked round-bottom flask was fitted with a Dewar condenser, N_2 inlet, rubber stopper, and a large egg-shaped magnetic stir bar [1]. The apparatus and the condenser were cooled with dry ice/isopropanol baths and flushed with nitrogen. Ammonia (80 mL) was condensed into the flask. Dry ether (45 mL) and naphthalene (5.00 g, 39 mmol) were added with stirring. Sodium metal (0.38 g) was added in three pieces over a five-minute period. The blue solution was stirred for 30 minutes and quenched with saturated ammonium chloride (12 mL). The ammonia was allowed to evaporate, and the mixture was transferred to a separatory funnel where the organic layer was washed four times with ammonium chloride and dried over sodium sulfate. The organic layer was concentrated under reduced pressure to give a white solid (1.2 g, 24% yield of **34**, used without purification). ^{1}H NMR (400 MHz, $CDCl_3$) δ = 3.39 (d, 4H, J = 1.31 Hz), 5.92 (t, 2H, J = 1.48 Hz), 7.09–7.17 (AA′BB′, 4H).

*Preparation of 1,4-dihydro-2,3-epoxynaphthalen[2](**35**)*

A 100 mL 3-necked round-bottom flask was fitted with two stoppers, a dropping funnel, and a magnetic stir bar [2]. The flask was charged with 1,4-dihydronaphthalene (0.34 g) in of dichloromethane (15 mL) and cooled to 0 °C. *m*-CPBA (0.70 g) was dissolved in dichloromethane (15 mL) and added dropwise over a 5-minute period. The reaction was stirred for 5 hours then filtered directly into the separatory funnel. The lower organic layer was washed with saturated sodium bicarbonate (2 × 25 mL), water (2 × 25 mL), and brine (2 × 25 mL). The mixture was dried over sodium sulfate and concentrated under reduced pressure. The white solid was purified by column chromatography (60 mesh silica gel, 3:1 hexanes: ether) and **35** (0.376 g, 100% yield) was recovered as a white solid. ^{1}H NMR (400 MHz, $CDCl_3$) δ = 3.19 (A**B**, 2H, J = 19.04 Hz), 3.31 (**A**B, 2H, J = 19.04 Hz), 3.47 (app s, 2H), 7.02–7.07 (AA′**BB′**, 2H), 7.12–7.17 (**AA′**BB′, 2H).

*Preparation of 1-bromo-1,2-dihydro--2,3-epoxynaphthalene (**36**)*

An oven-dried 100-mL 3-necked round-bottom flask was fitted with two stoppers, a reflux condenser and magnetic stir bar. The flask was charged with **35** (0.14 g, 0.9 mmol) and NBS (0.27 g) in dry dichloromethane (12 mL). AIBN (0.021 g) was dissolved in dichloromethane (8 mL) and added to the reaction flask. The mixture was heated at gentle reflux for 51 hours. The lower organic layer was washed with water (2 × 50 mL) and brine (2 × 50 mL), dried over sodium sulfate, and concentrated under reduced pressure to give a yellow oil/solid. The crude product was purified by column chromatography (60 mesh silica gel, 3:1 hexanes:ether) and the first fraction was collected to give a 46% yield of **36** (99 mg) as white needles. ^{1}H NMR (400 MHz, $CDCl_3$) δ = 3.27 (A**B**, 1H, J = 17.55 Hz), 3.31 (**A**B, 1H, J = 17.55 Hz), 3.62–3.66 (m, 1H), 3.76–3.80 (m, 1H), 5.54 (d, 1H, J = 3.67), 7.08-7.11 (m, 1H), 7.21–7.31 (m, 3H).

*Preparation of 4,5-benzoxepin via dehydrohalogenation[2] (**11**)*

An oven-dried 100-mL 3-necked round-bottom flask was fitted with N_2 inlet, magnetic stir bar, and dropping funnel. The flask was charged with potassium t-butoxide (0.224 g, 4 eq) in 20 mL of dry TFH and cooled to 0 °C. **36** (0.114 g, 1eq) was dissolved in 5 mL of dry THF and added dropwise over a 50-minute period. After addition, the funnel was replaced with a calcium chloride drying tube. After 24 hours the mixture was filtered and concentrated. The crude product was purified by column

chromatography (60 mesh silica gel, 3:1 hexanes:ether). The first fraction was collected as **11** (60 mg, 83% yield) as a yellow solid. ^{1}H NMR (400 MHz, $CDCl_3$) δ = 5.06 (dd, 2H, J = 7.70, 3.99 Hz), 5.67 (dd, 2H, J = 7.38, 3.98 Hz), 6.61–6.66 (AA′**XX′**, 2H), 6.88-6.92 (**AA′**XX′, 2H). ^{13}C NMR (100 MHz, $CDCl_3$) δ = 146.2, 136.0, 129.2, 127.9, 113.4. EI-MS (DCM) = 144 (85), 115 (100), 89 (35). UV-Vis (0.2 mg/mL in DCM) λ_{max} = 310.2 nm.

2. Characterization

2.1. Enzyme Incubations with 4,5-benzoxepin

Below are the total ion chromatograms for the reactions of 4,5-benzoxepin with P450 isoforms and pHLM followed by corresponding EI-MS.

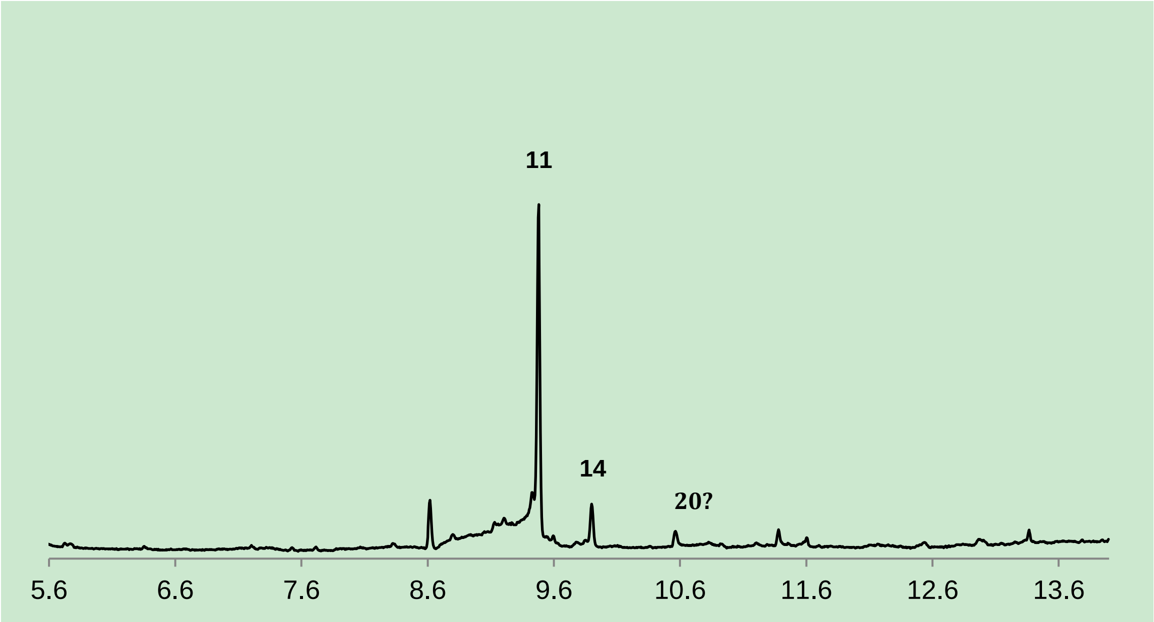

Figure S1. Reaction of **11** with P450 2E1.

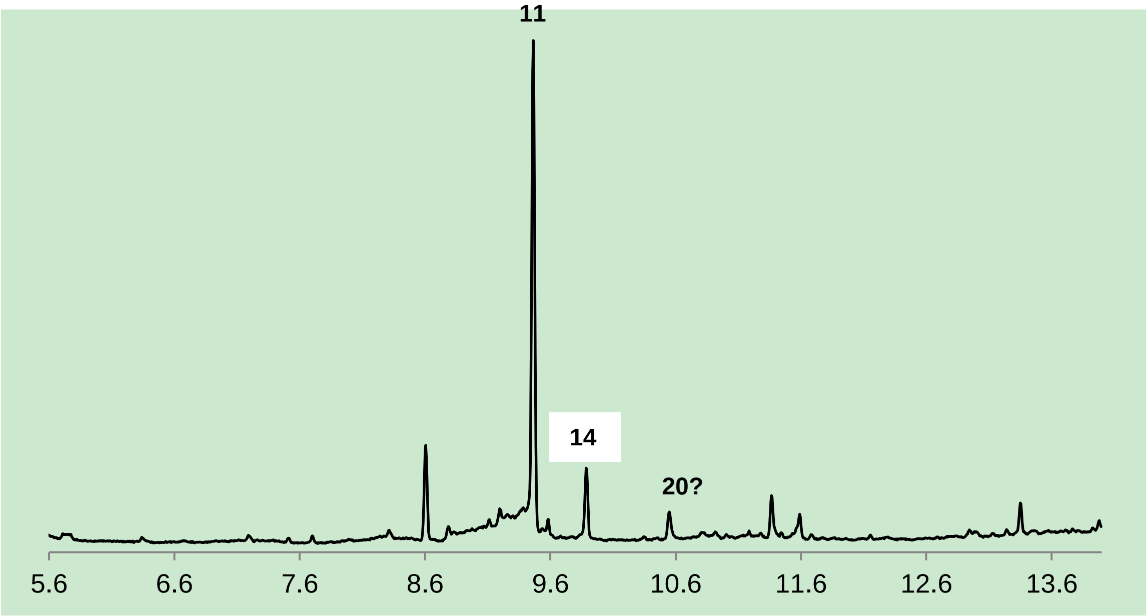

Figure S2. Reaction of **11** with P450 3A4.

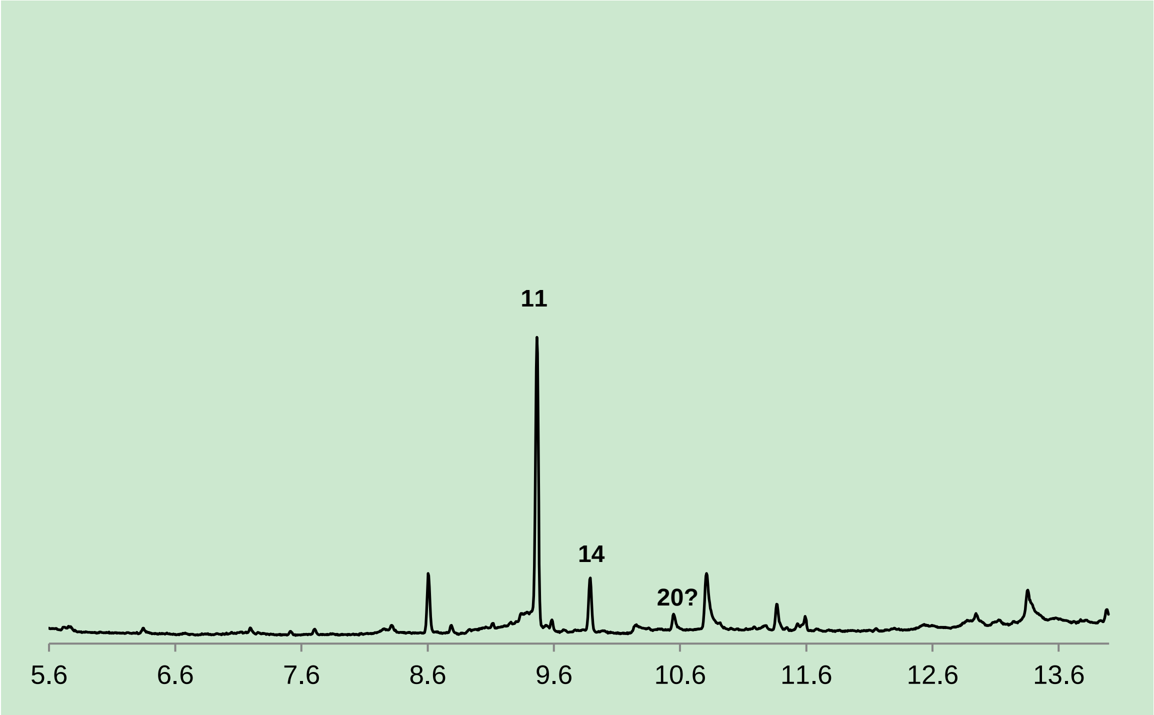

Figure S3. Reaction of **11** with pHLM.

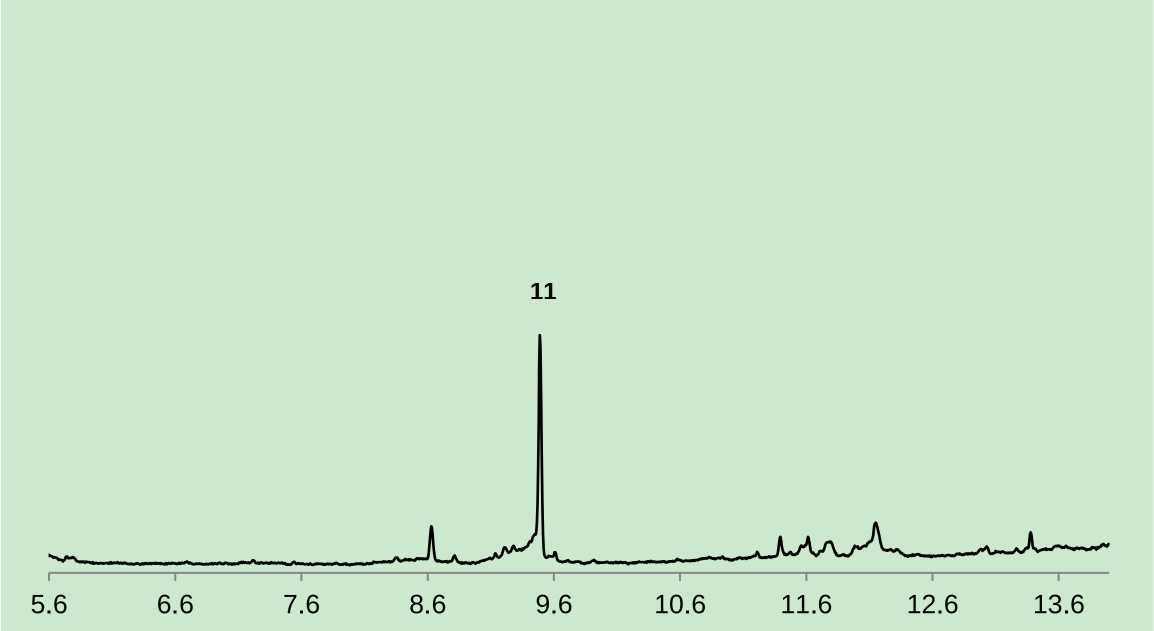

Figure S4. Incubation of **11** with no enzyme—control experiment.

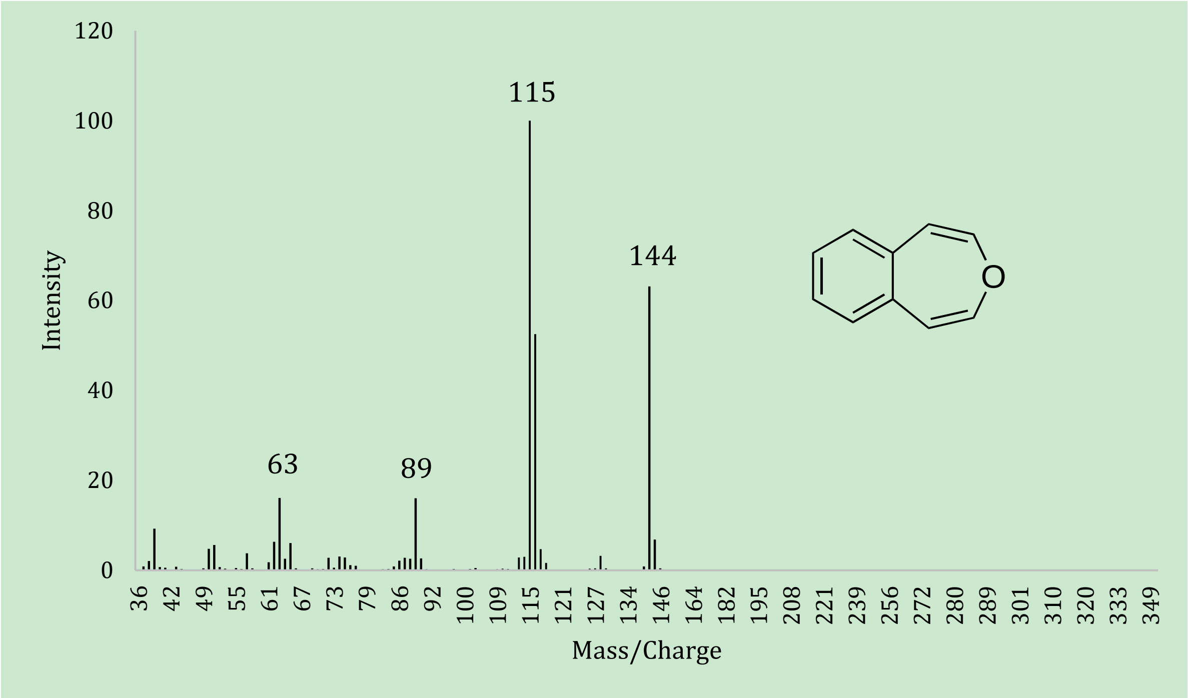

Figure S5. EI-MS of **11** (retention time = 9.4 min).

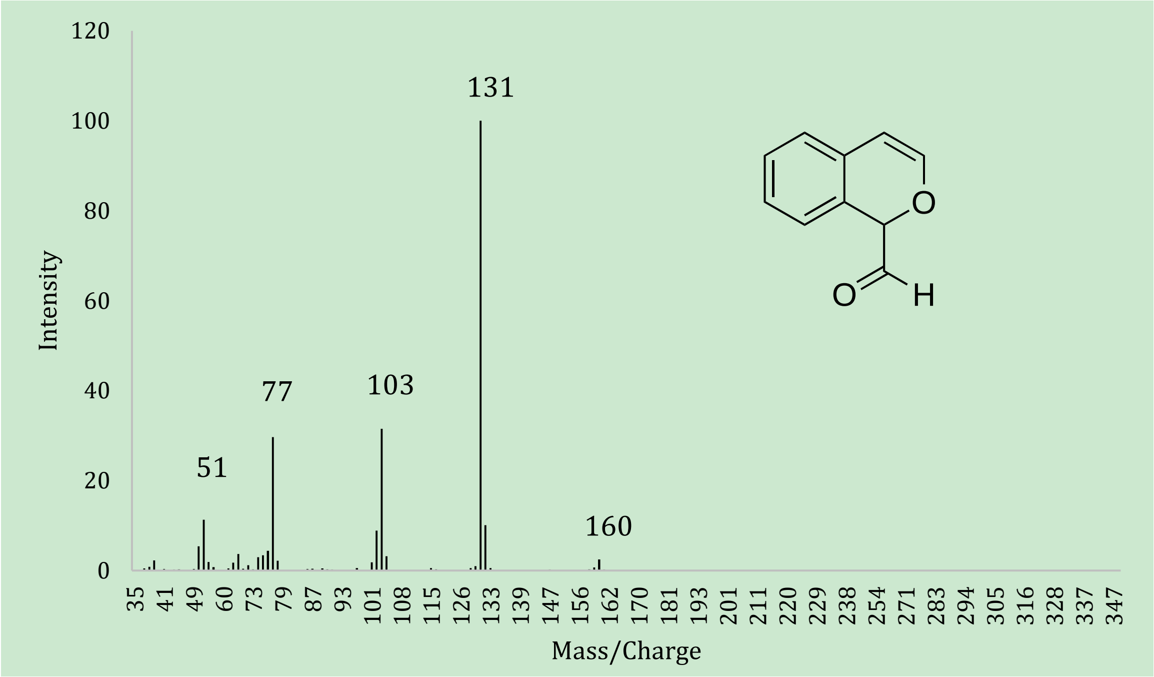

Figure S6. EI-MS of 14 (retention time = 9.8 min).

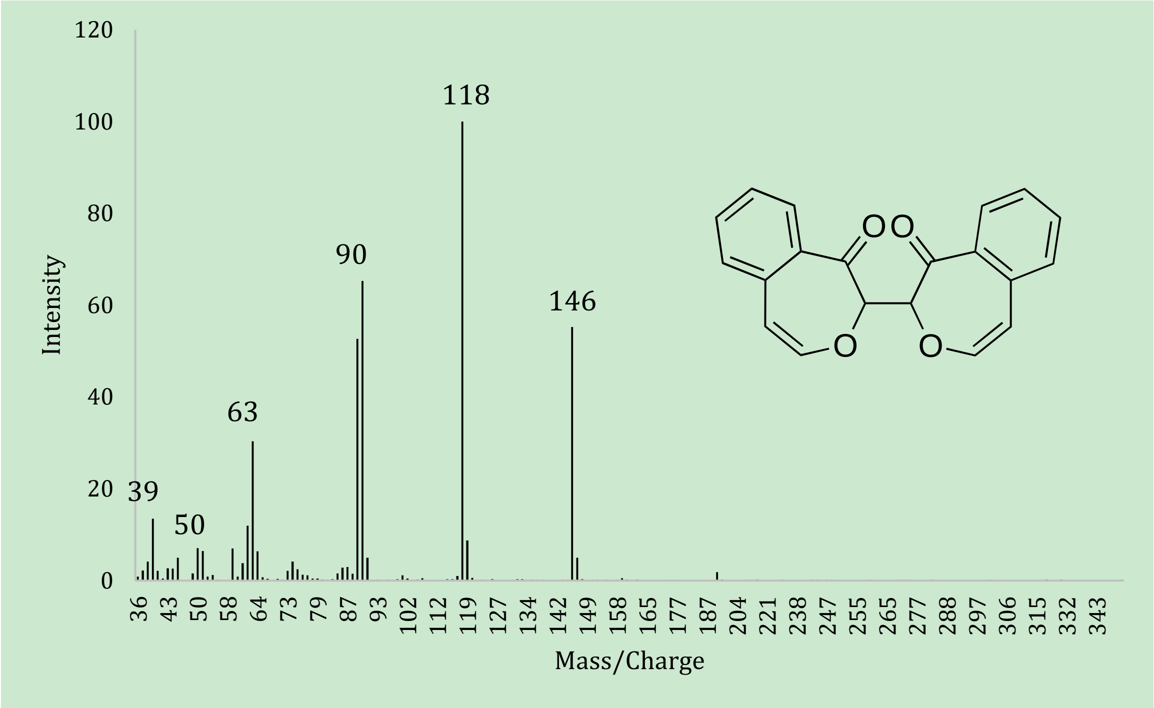

Figure S7. EI-MS of **20** (retention time = 10.5 min).

2.2. Enzymatic Incubations with 2,7-dimethyloxepin

Below are the total ion chromatograms for the reactions of 4,5-benzoxepin with P450 isoforms and pHLM followed by corresponding EI-MS.

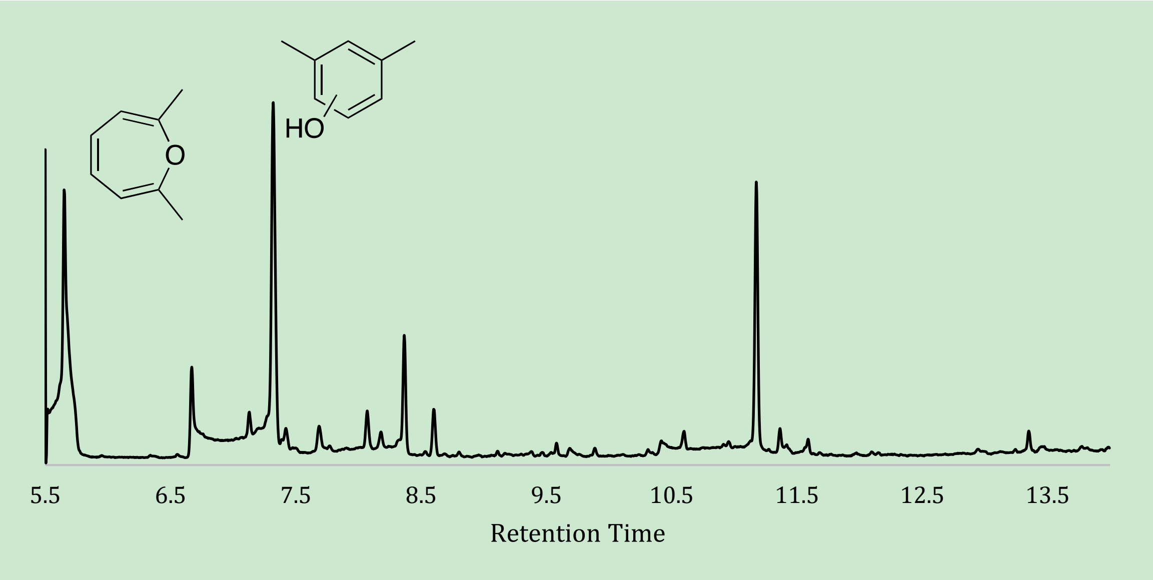

Figure S8. Incubation of 2,7-dimethyloxepin **8** with P450 1A2.

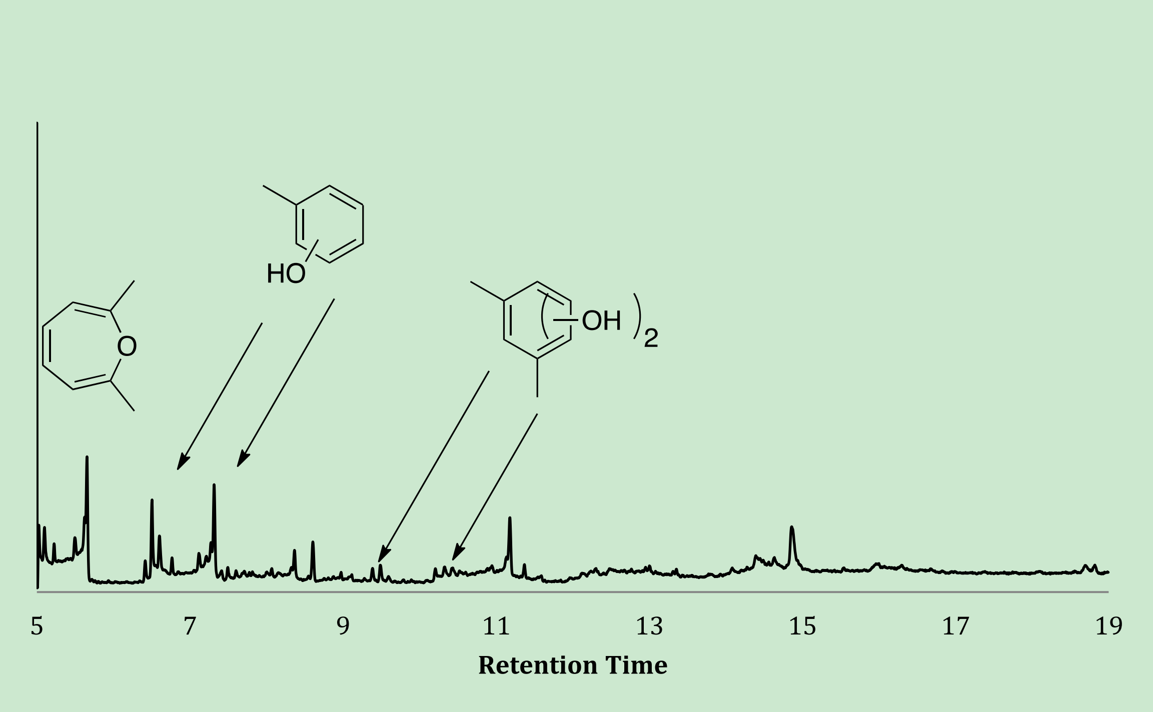

Figure S9. Incubation of **8** with P450 3A4.

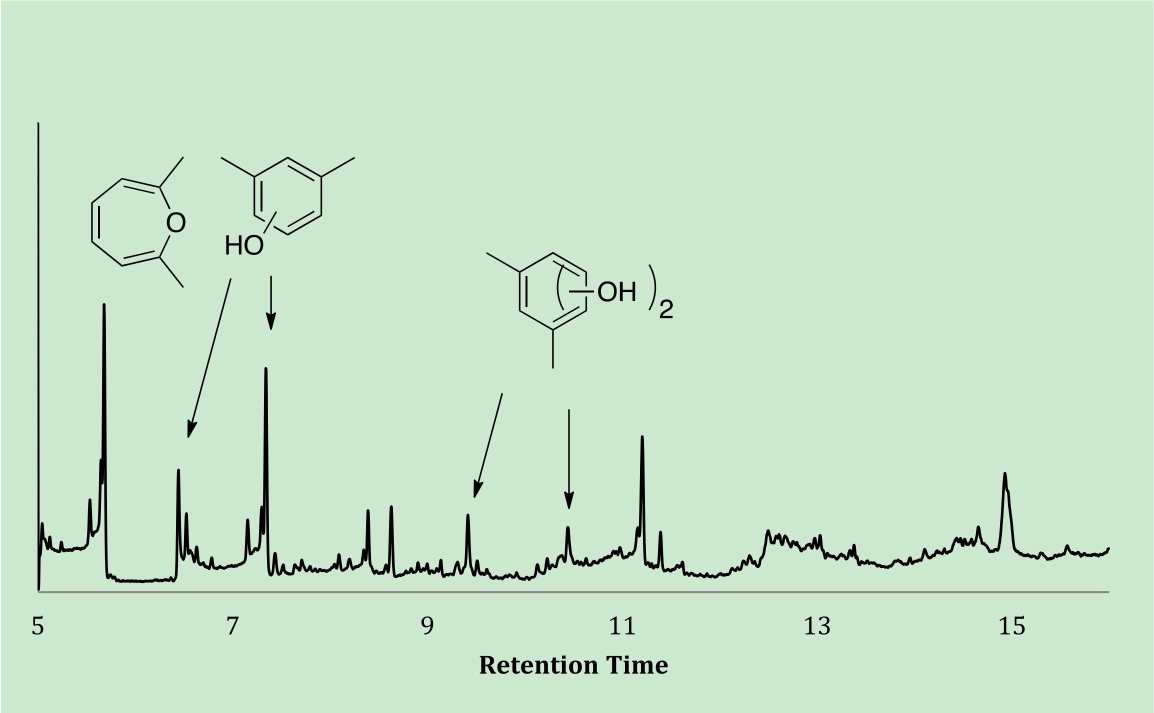

Figure S10. Incubation of **8** with pHLM.

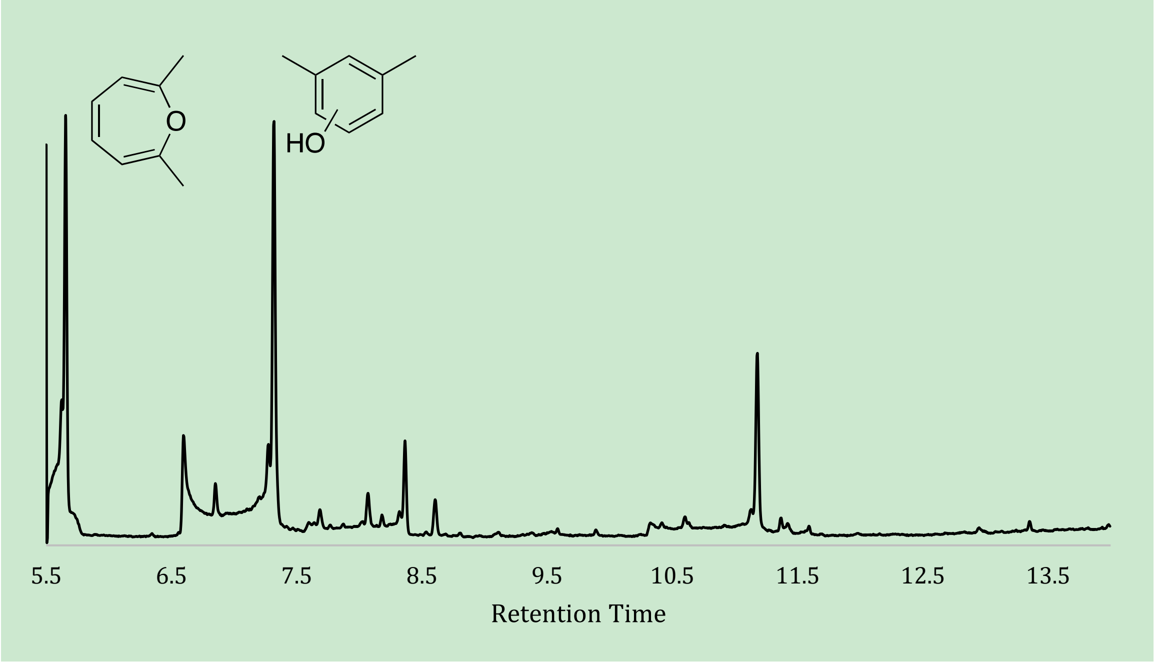

Figure S11. Incubation of 8 with P450 2E1.

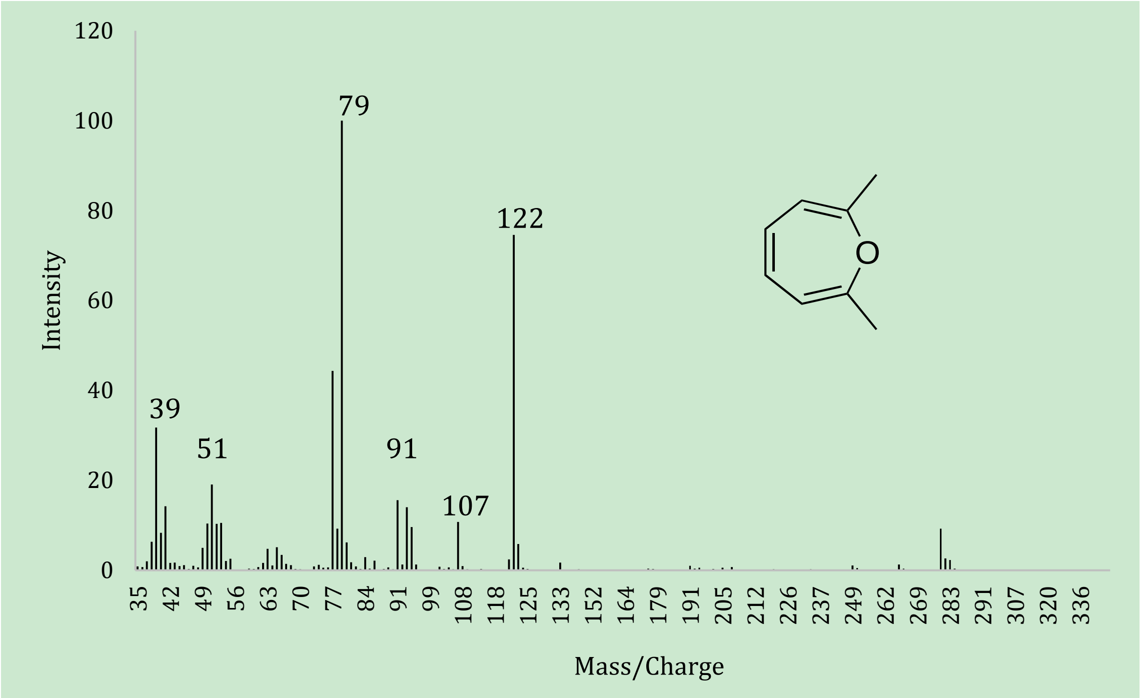

Figure S12. EI-MS dimethyloxepin (retention time = 5.6 min).

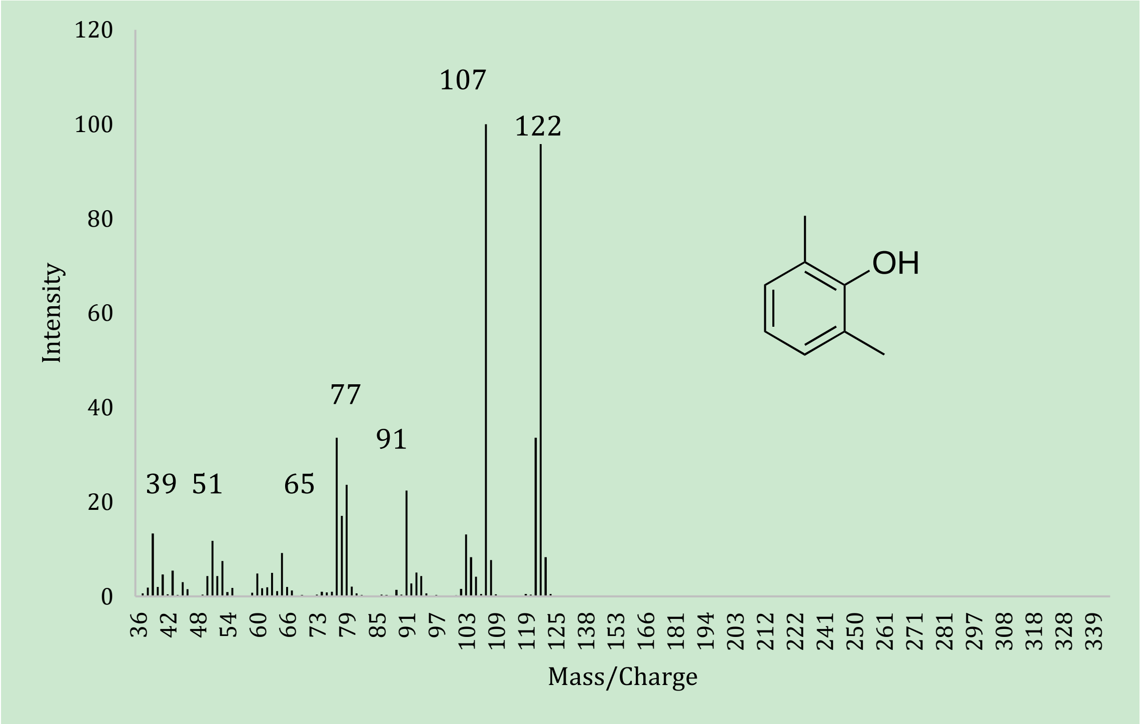

Figure S13. EI-MS dimethylphenol (retention time = 7.3 min).

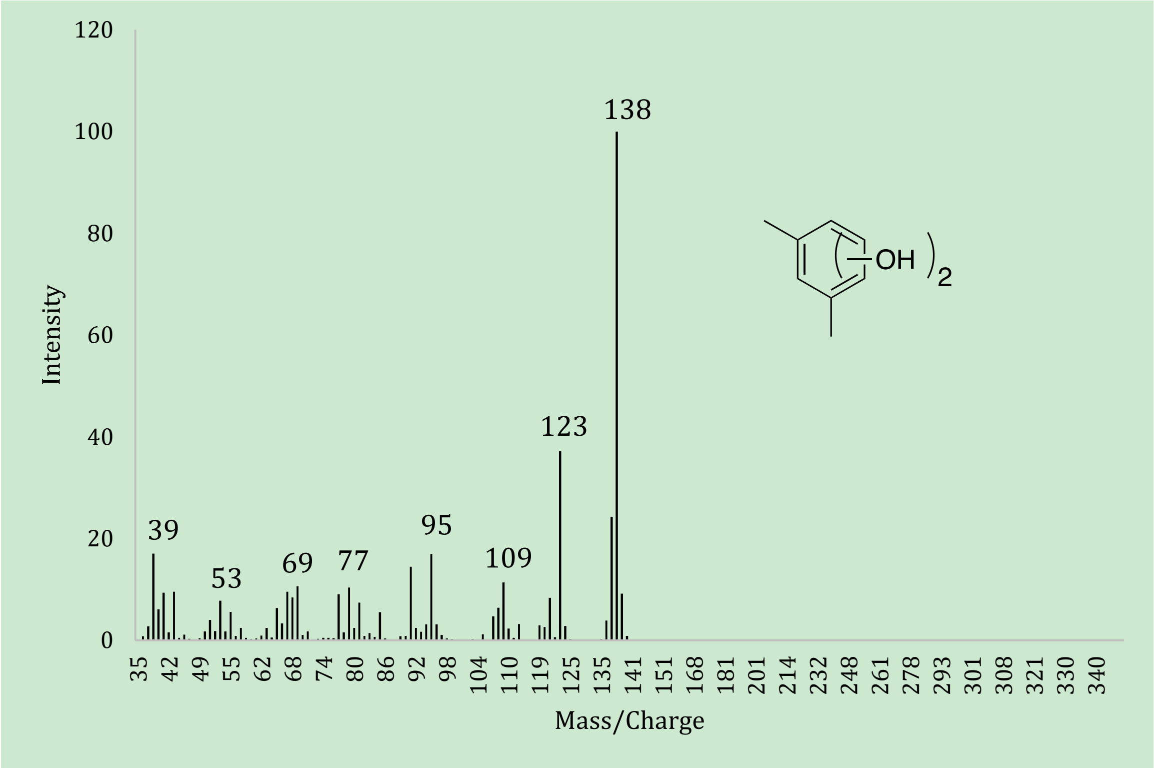

Figure S14. EI-MS dihydroxy-dimethylphenol (retention time = 9.4 min).

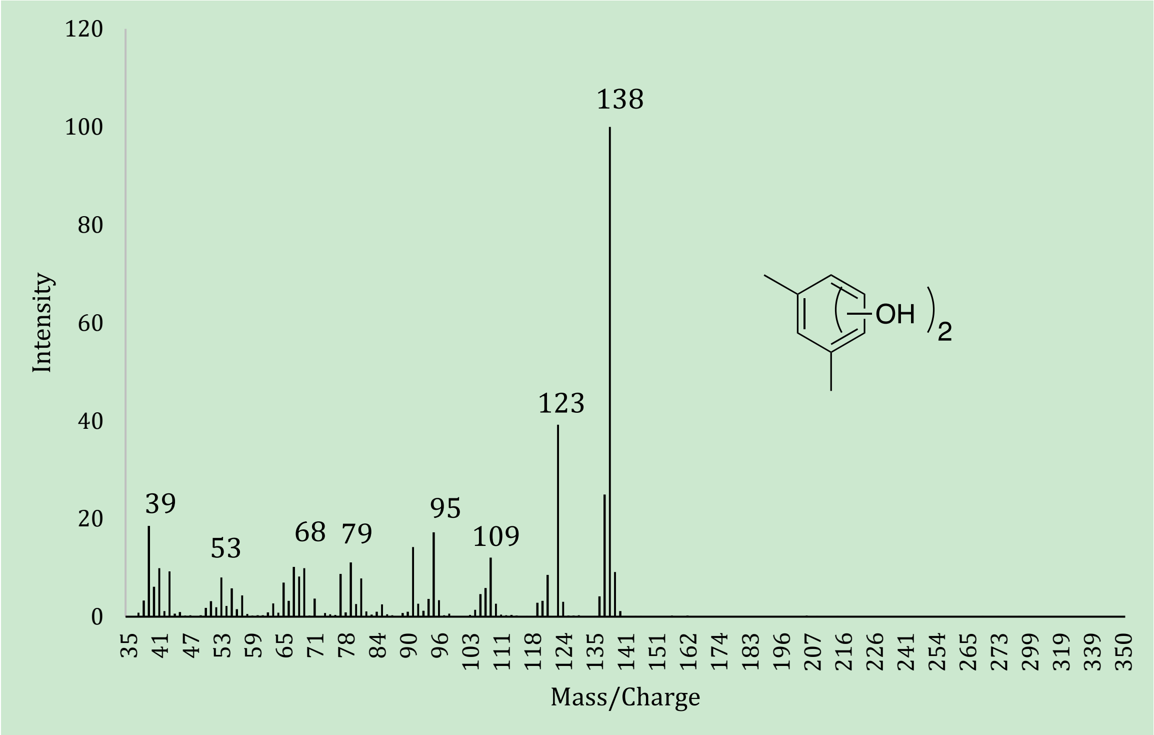

Figure S15. EI-MS dihydroxy-dimethylphenol (retention time = 10.4 min).

2.3. Synthesis of 4,5-benzoxepin—NMR data

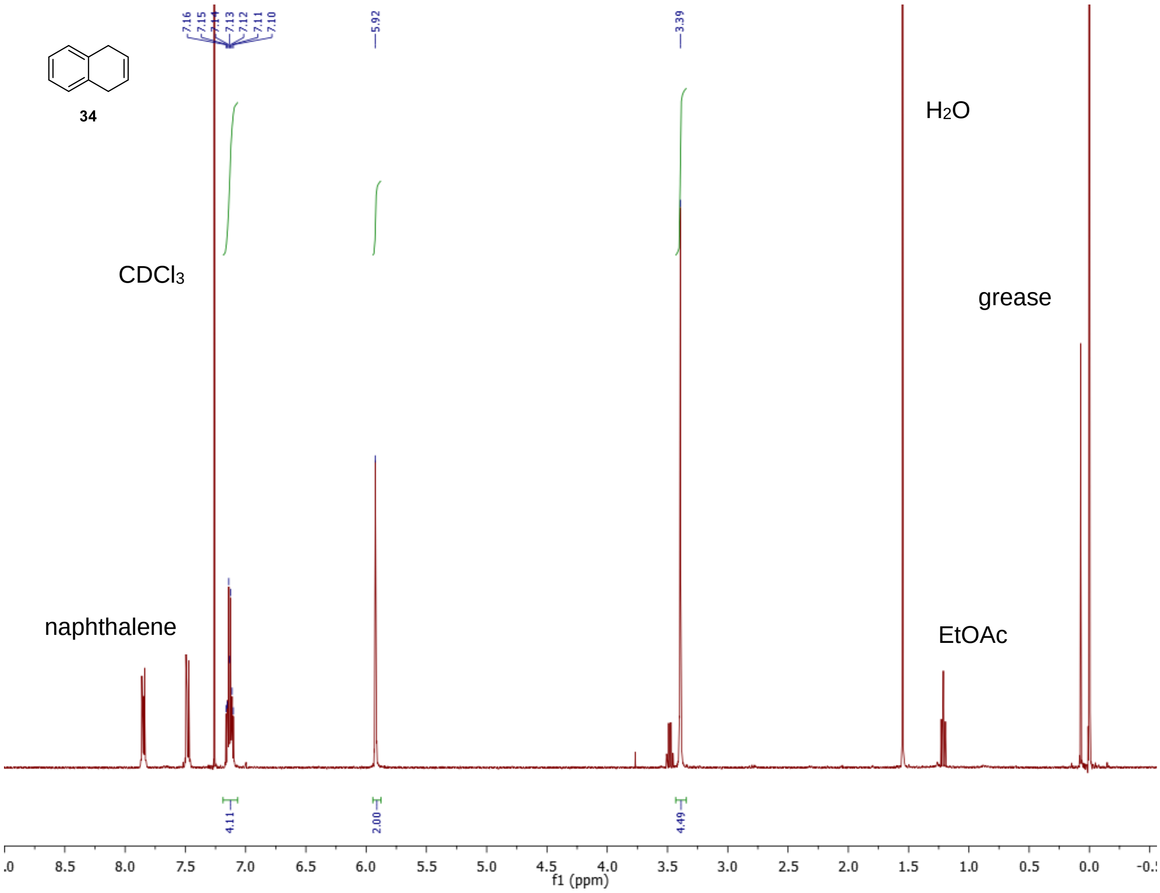

Figure S16. ^{1}H NMR (400 MHz, $CDCl_3$) of 1,4-dihydronaphthalene.

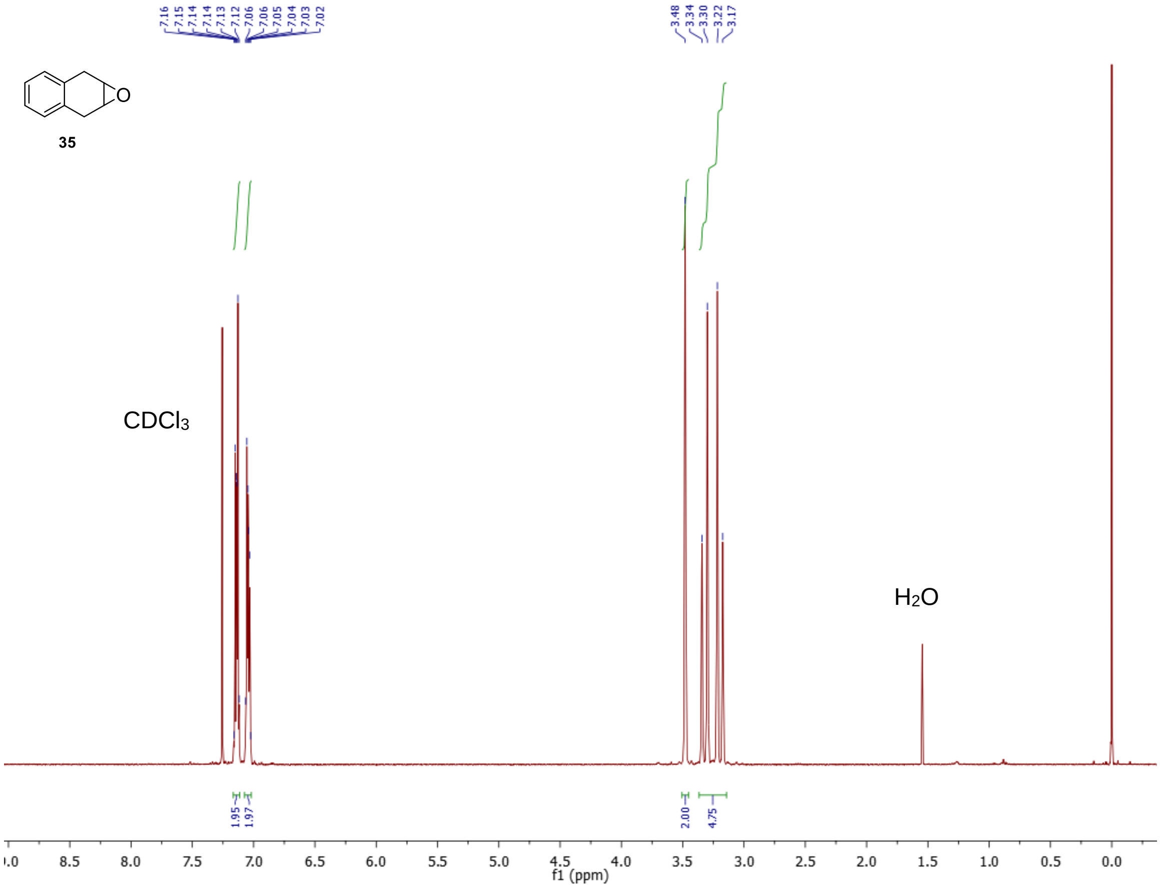

Figure S17. ^{1}H NMR (400 MHz, $CDCl_3$) of 1,4-dihydro-2,3-epoxynaphthalene.

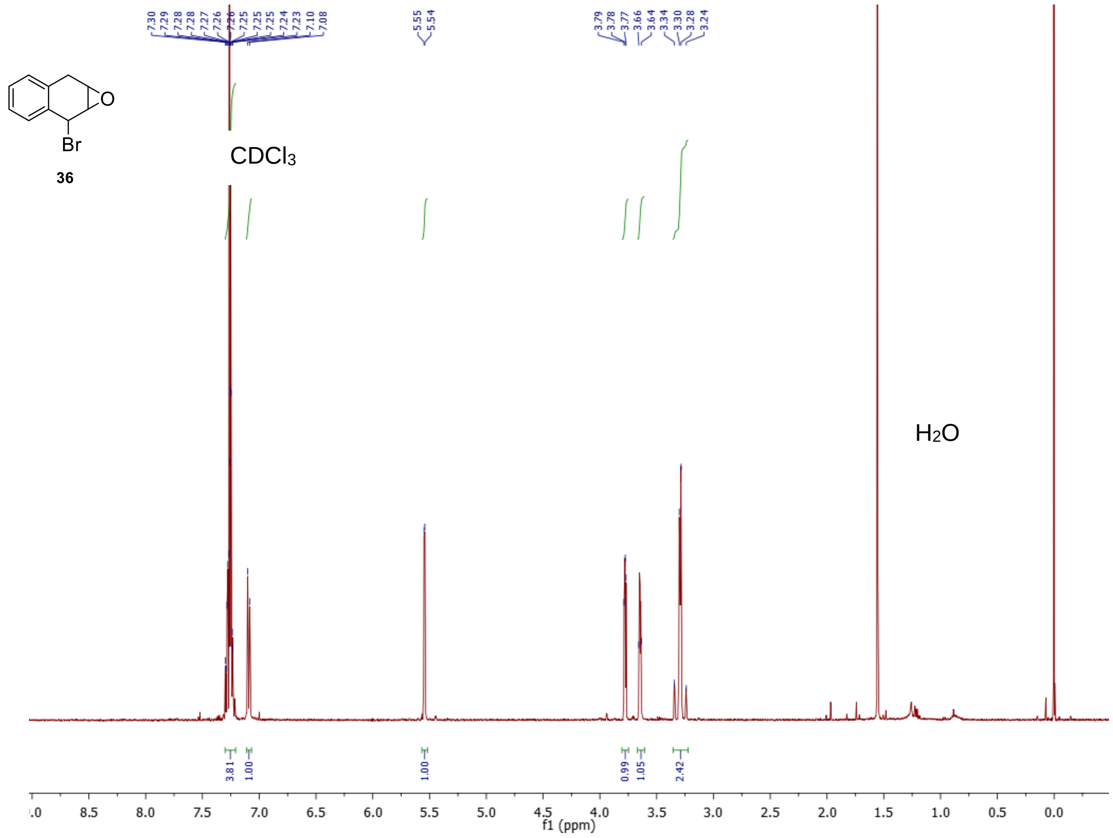

Figure S18. ^1H NMR (400 MHz, $CDCl_3$) of 1-bromo-1,2-dihydro--2,3-epoxynaphthalene.

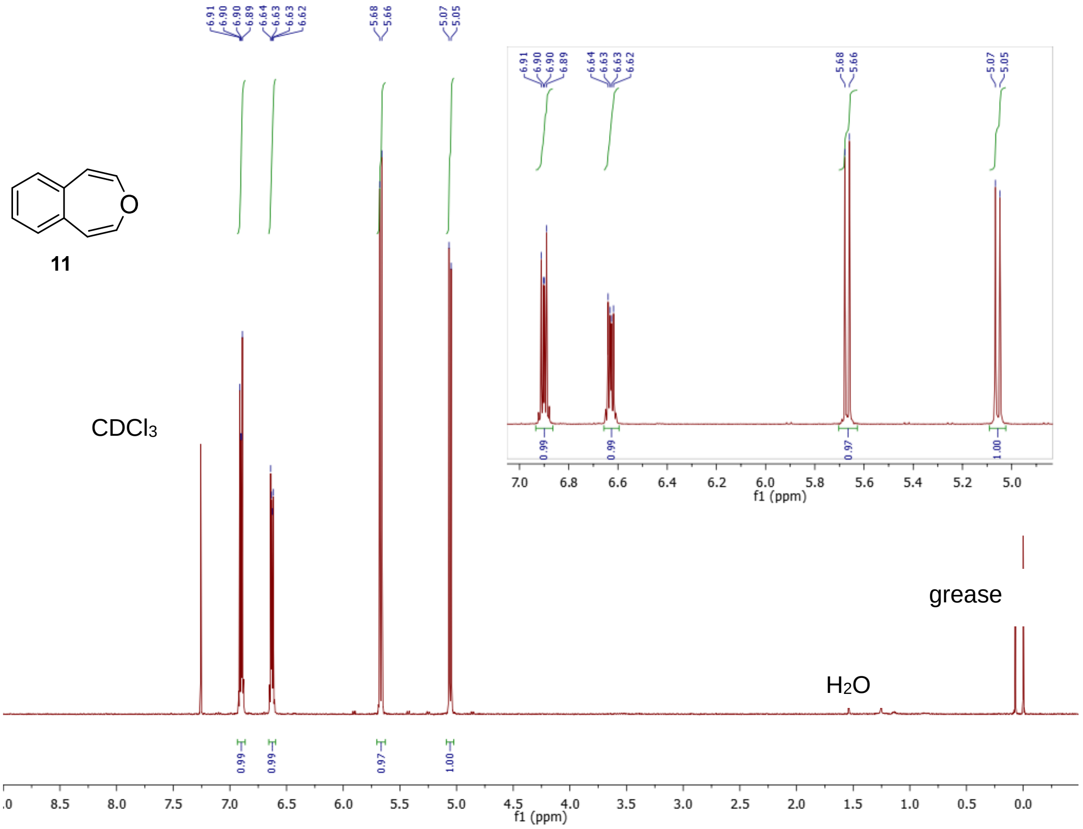

Figure S19. ^1H NMR (400 MHz, $CDCl_3$) of 4,5-benzoxepin.

2.4. Characterization of **20**

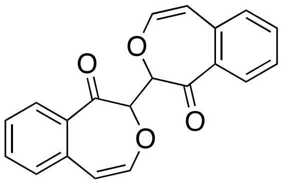

20

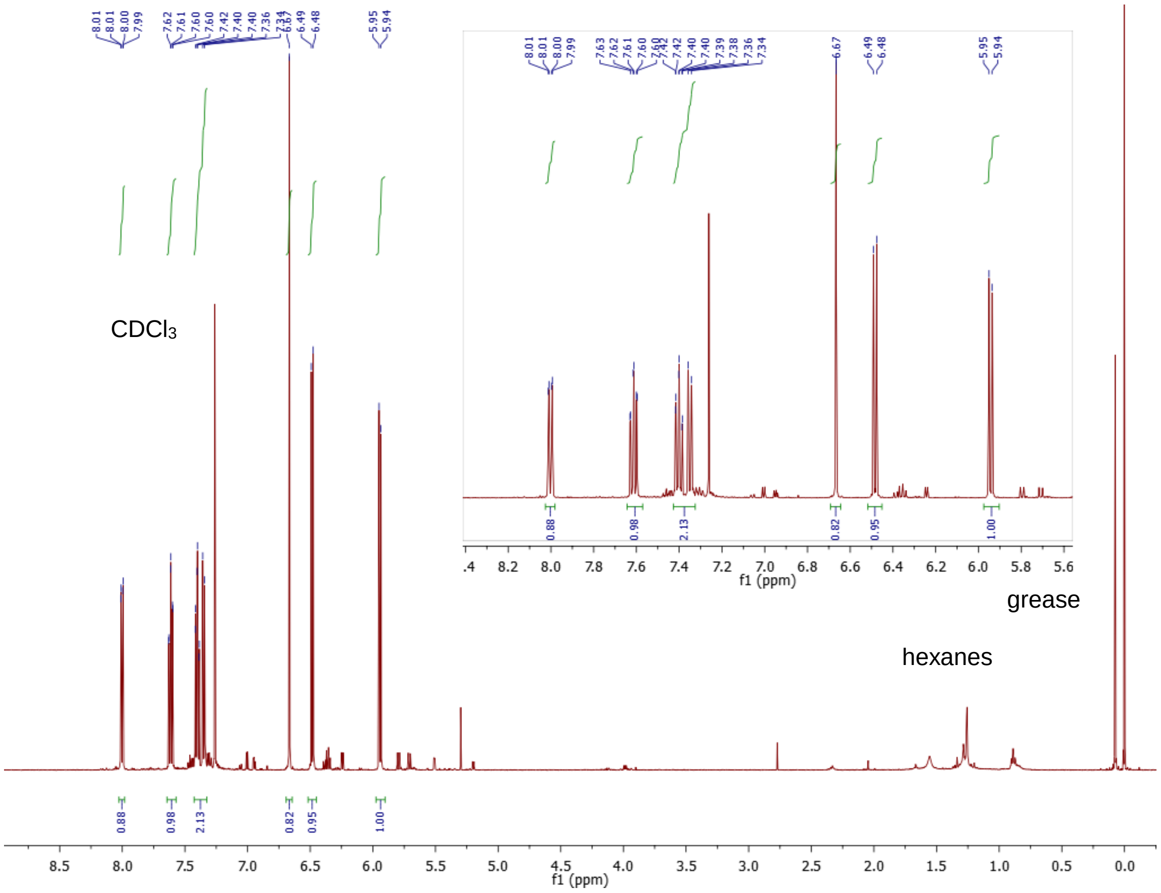

Figure S20. ^{1}H NMR (500 MHz, $CDCl_3$) of **20**.

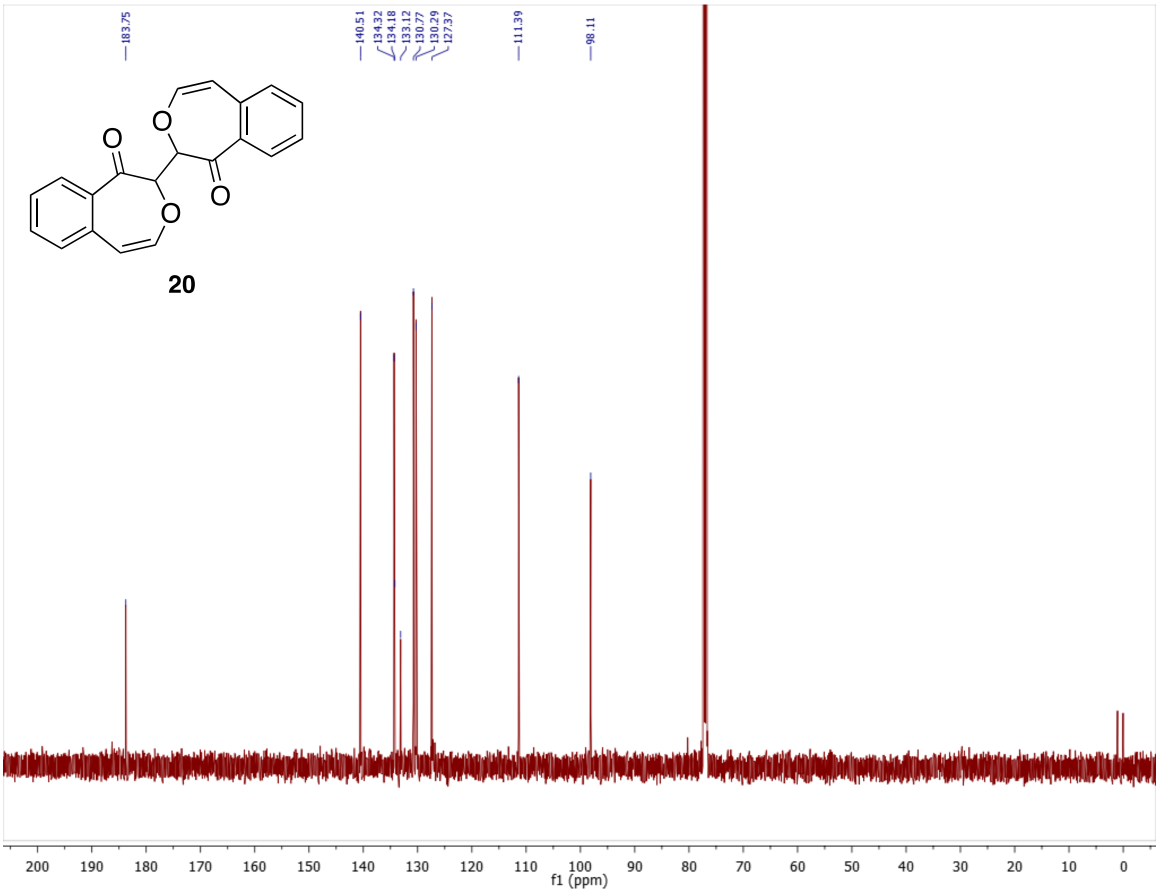

Figure S21. ^{13}C NMR (125 MHz, $CDCl_3$) of **20**.

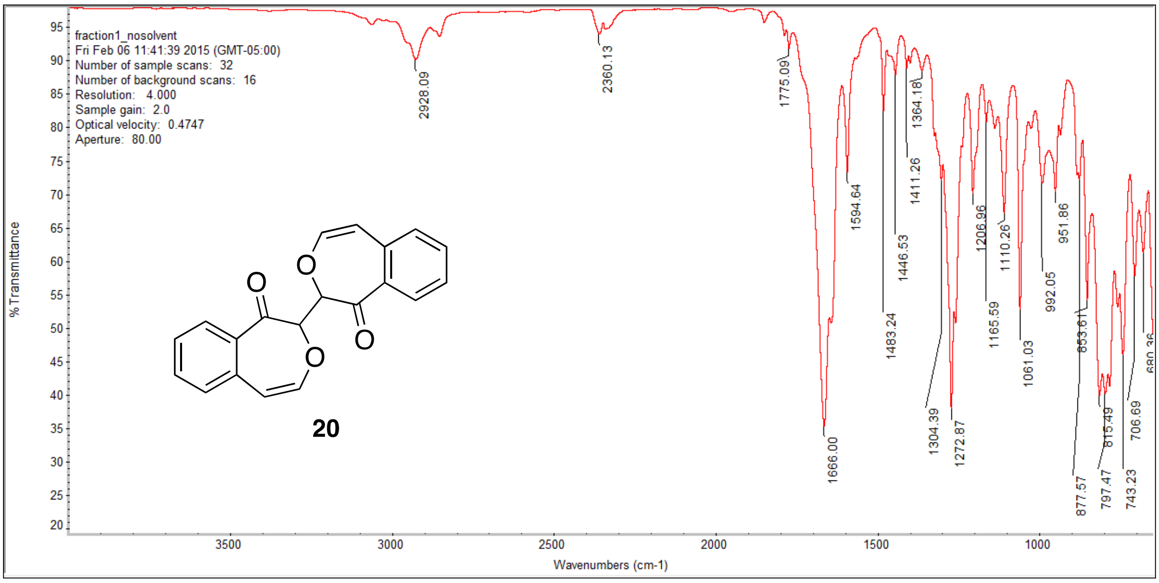

Figure S22. IR (neat) spectrum of **20**.

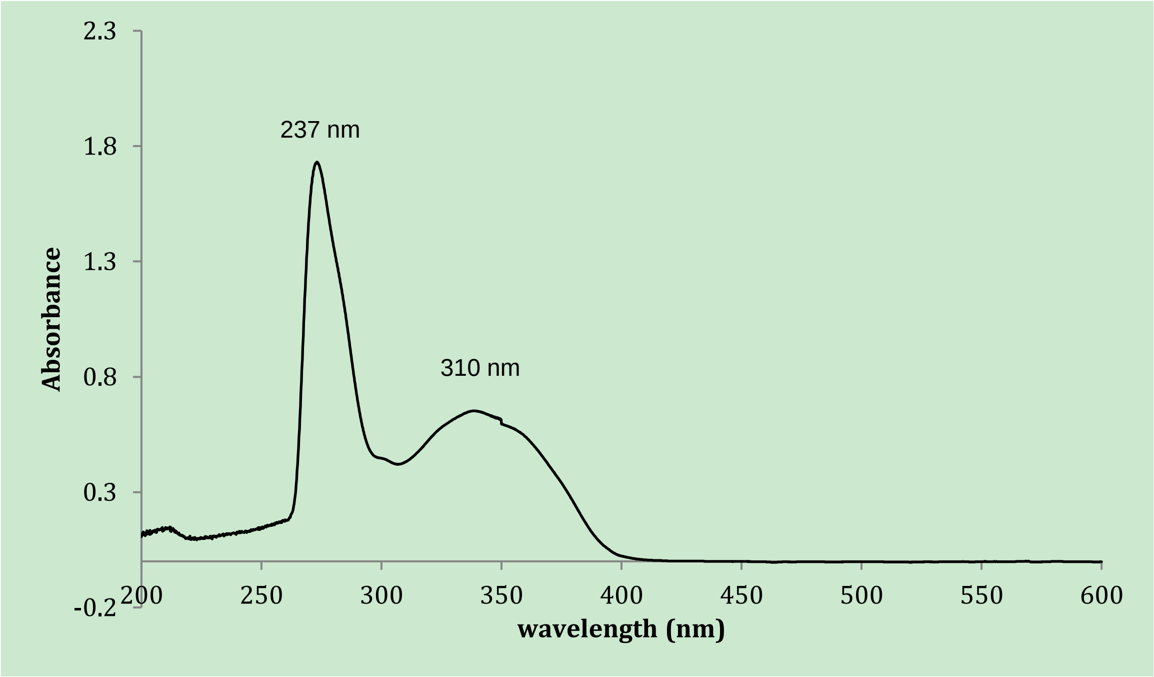

Figure S23. UV-Vis (0.2 mg/mL in dichloromethane) of **20**.

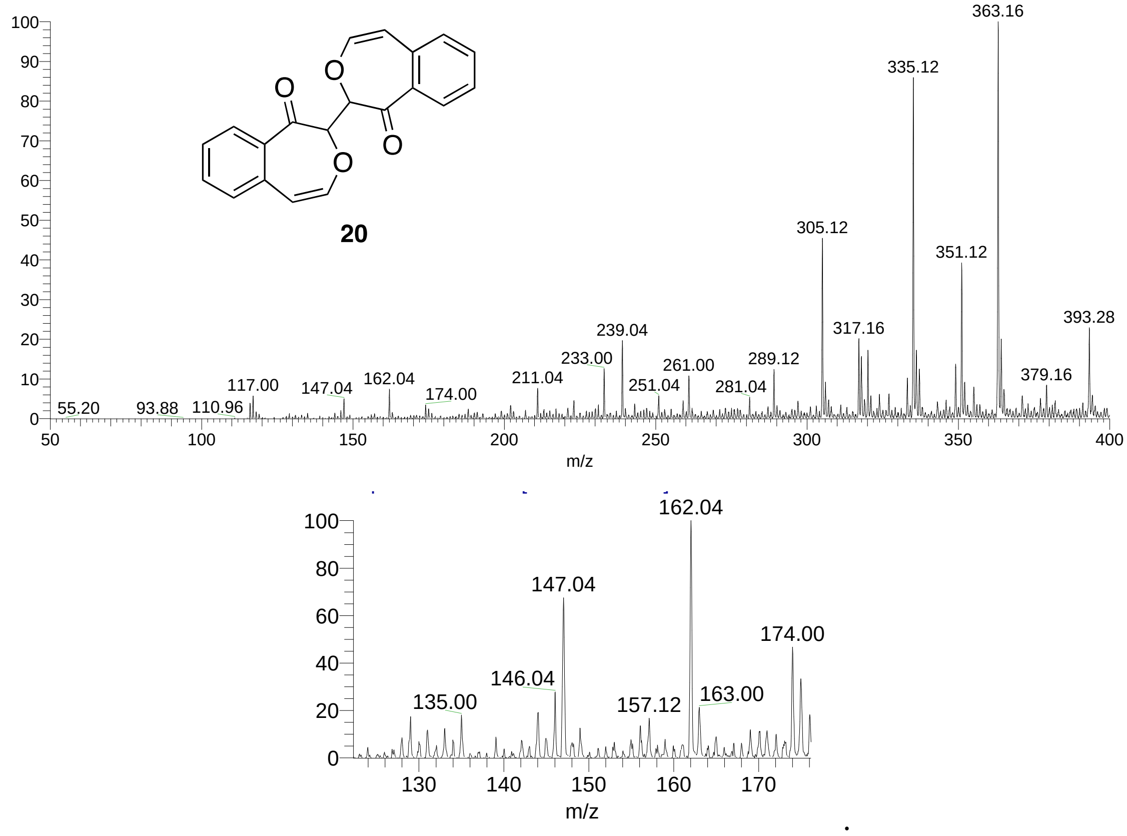

Figure S24. ESI-MS of 20.

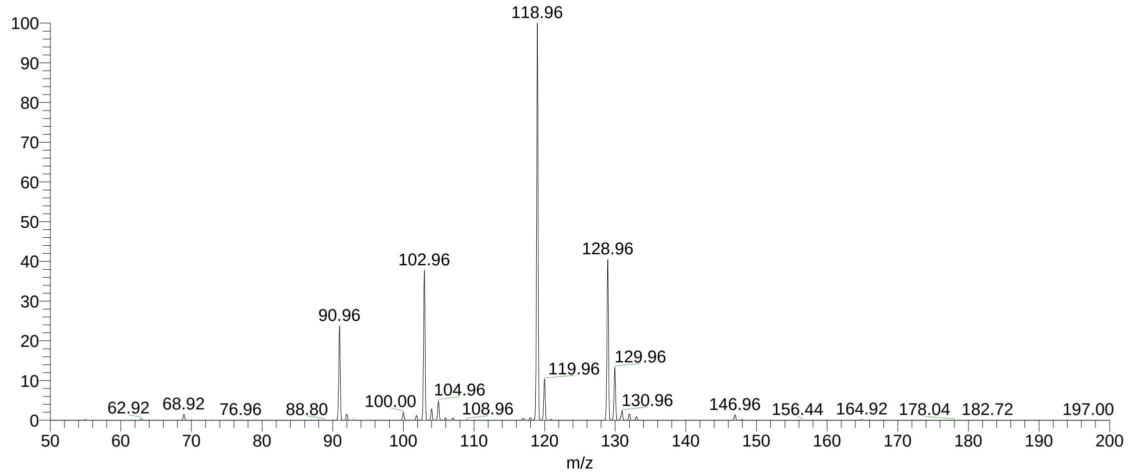

Figure S25. MS^2 of m/z 147.

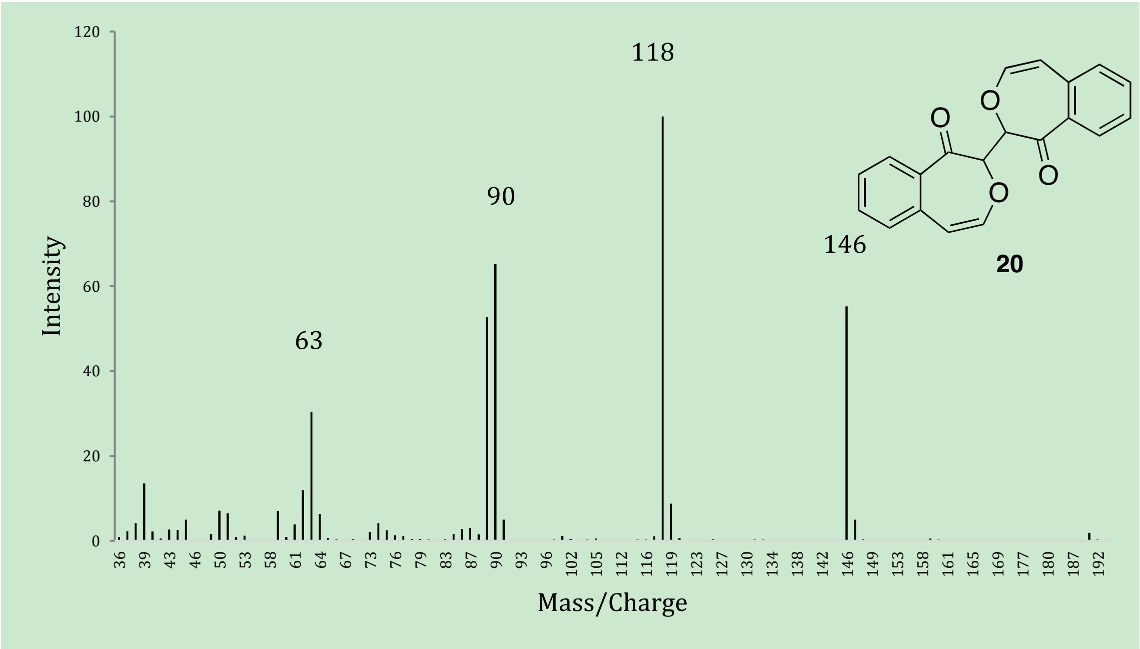

Figure S26. EI-MS of 20.

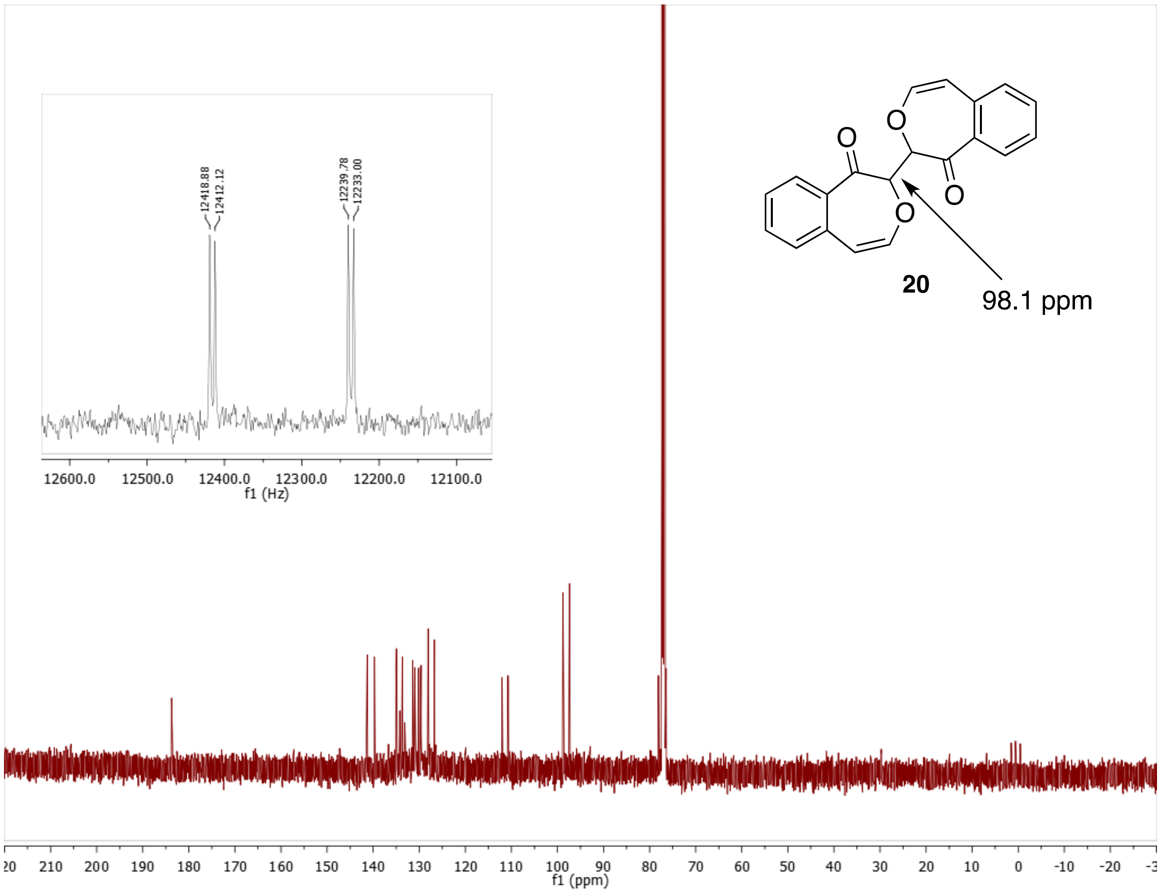

Figure S27. ^{13}C {^{1}H} coupled NMR (125 MHz, $CDCl_3$) of **20**.

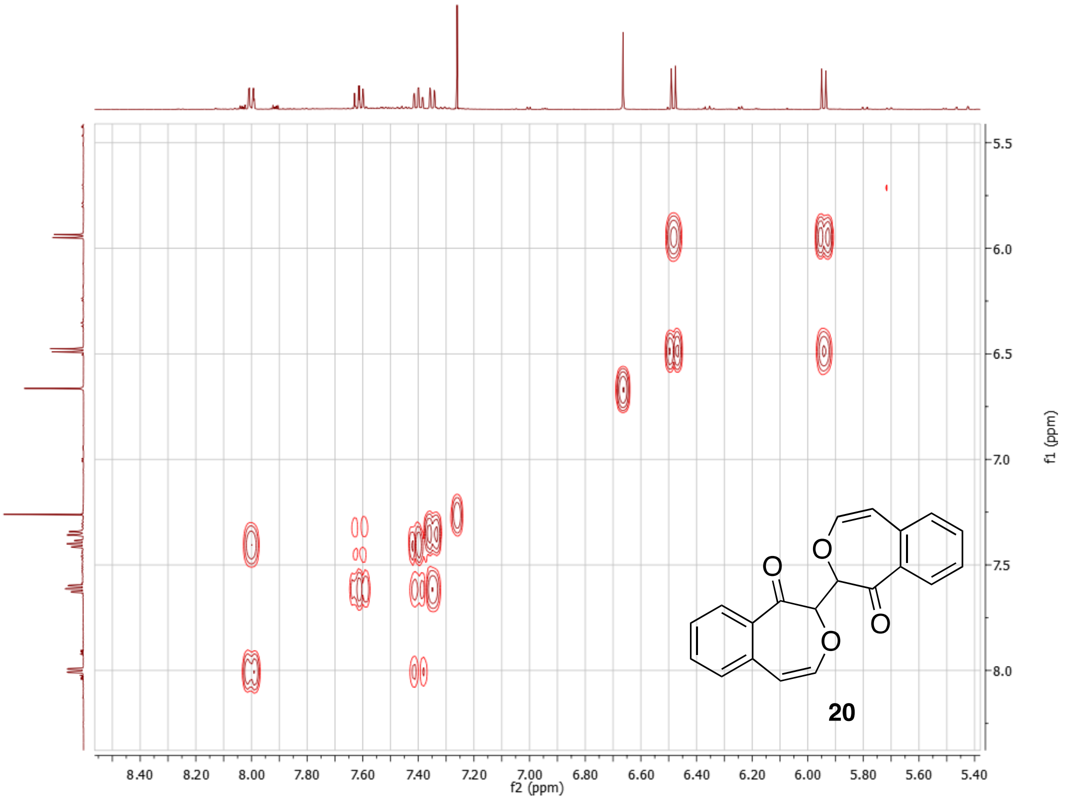

Figure S28. COSY (500 MHz, $CDCl_3$) of **20**.

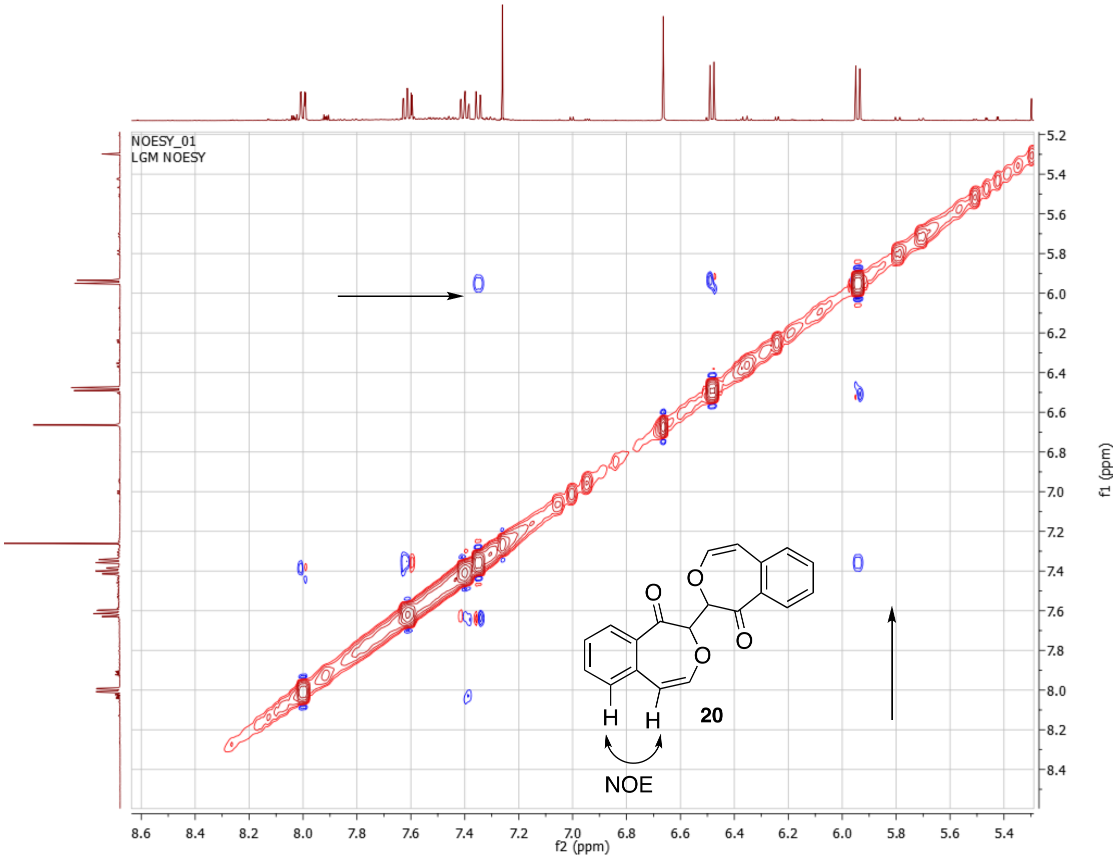

Figure S29. NOESY (500 MHz, $CDCl_3$) of **20**.

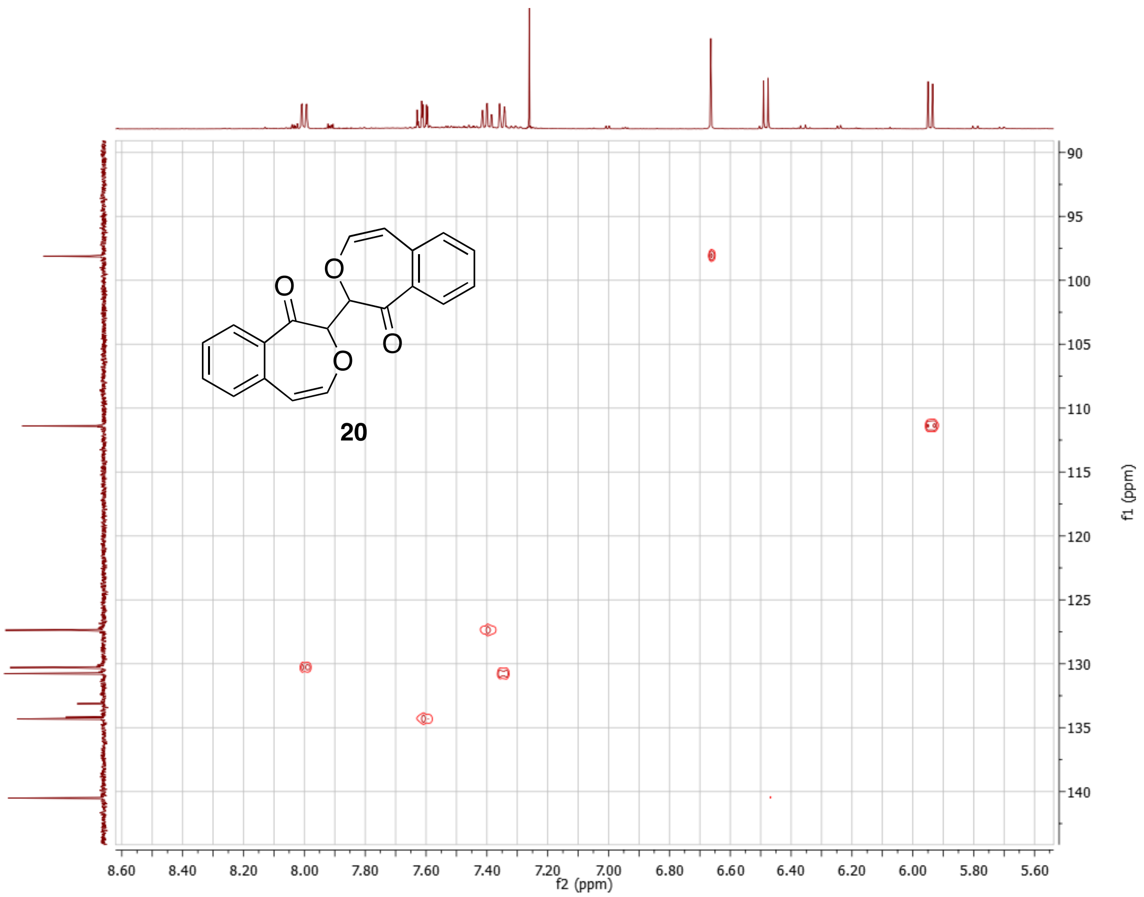

a)

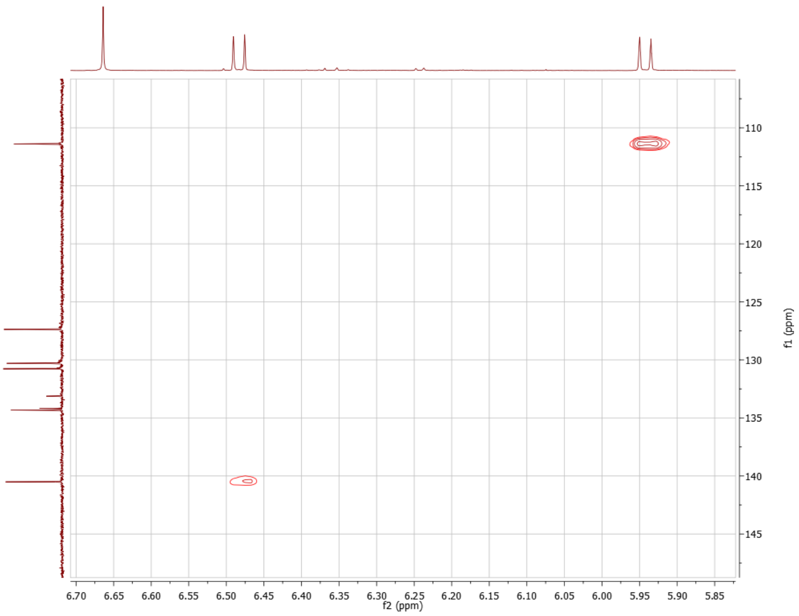

b)

Figure S30. a) HMQC (400 MHz, $CDCl_3$) of **20**; **b**) expansion of 5.85–6.7 ppm region.

2.5. Characterization of putative **21**.

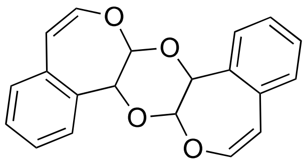

21

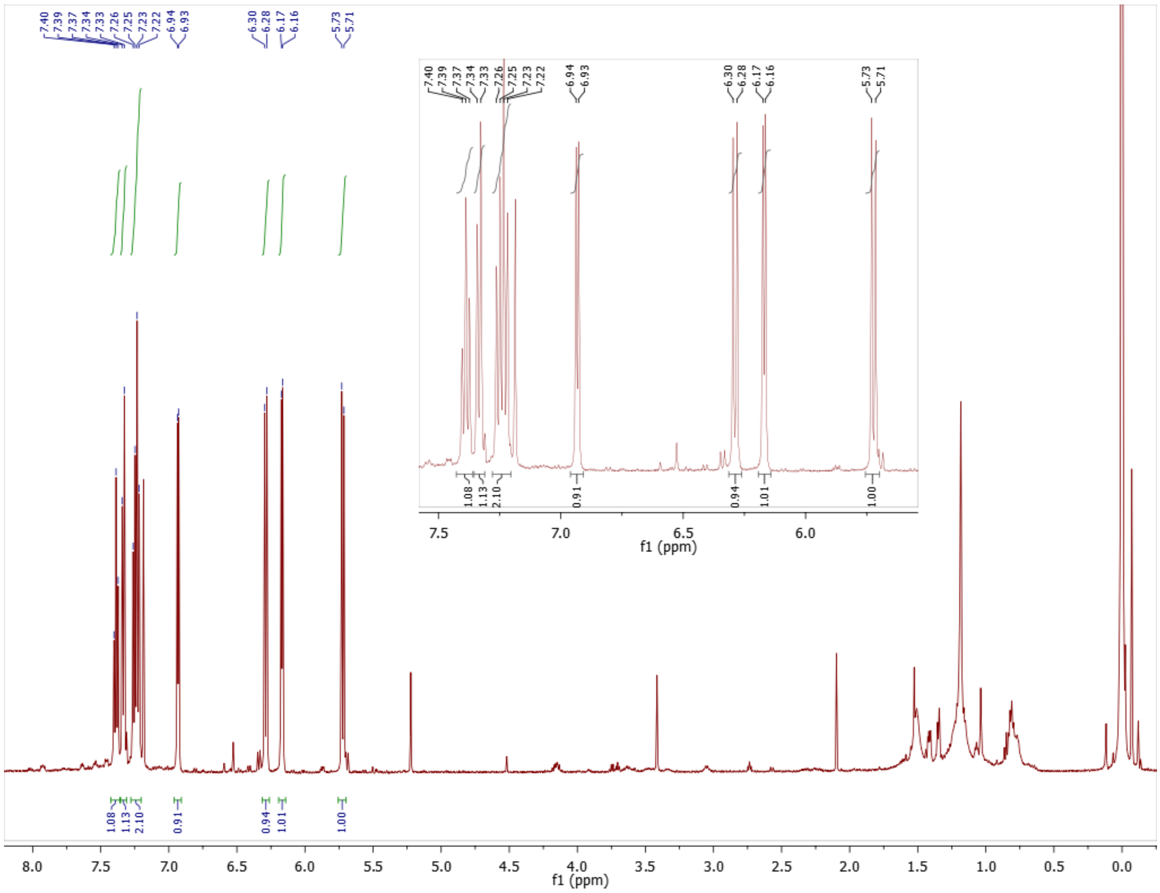

Figure S31. ^{1}H NMR (400 MHz, $CDCl_3$) of **21**.

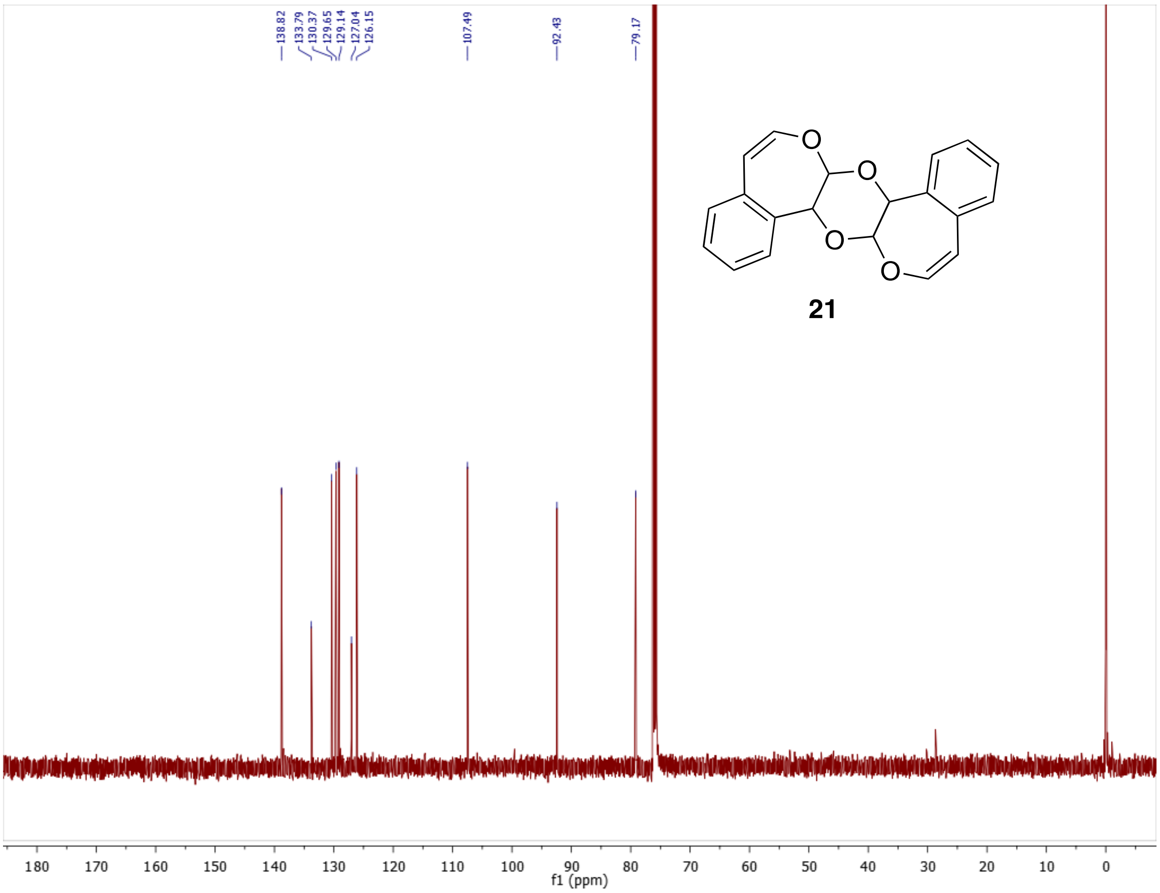

Figure S32. ^{13}C NMR (125 MHz, $CDCl_3$) of **21**.

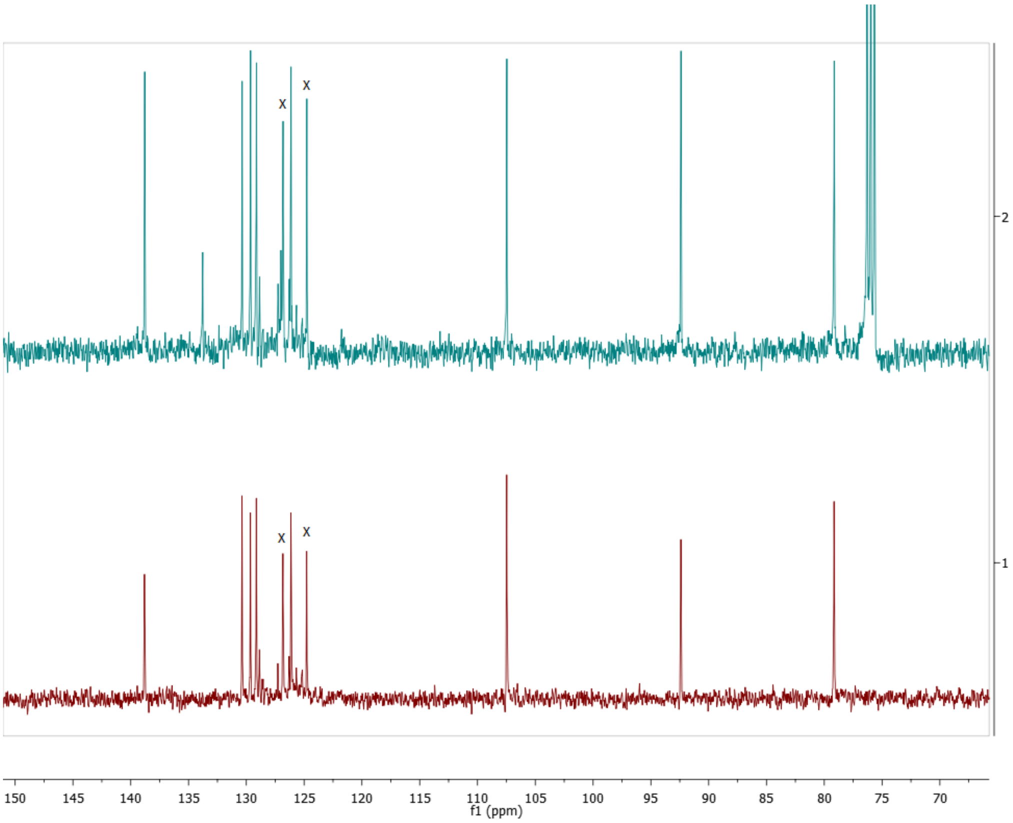

Figure S33. ^{13}C NMR and DEPT (125 MHz, $CDCl_3$) of **21**.

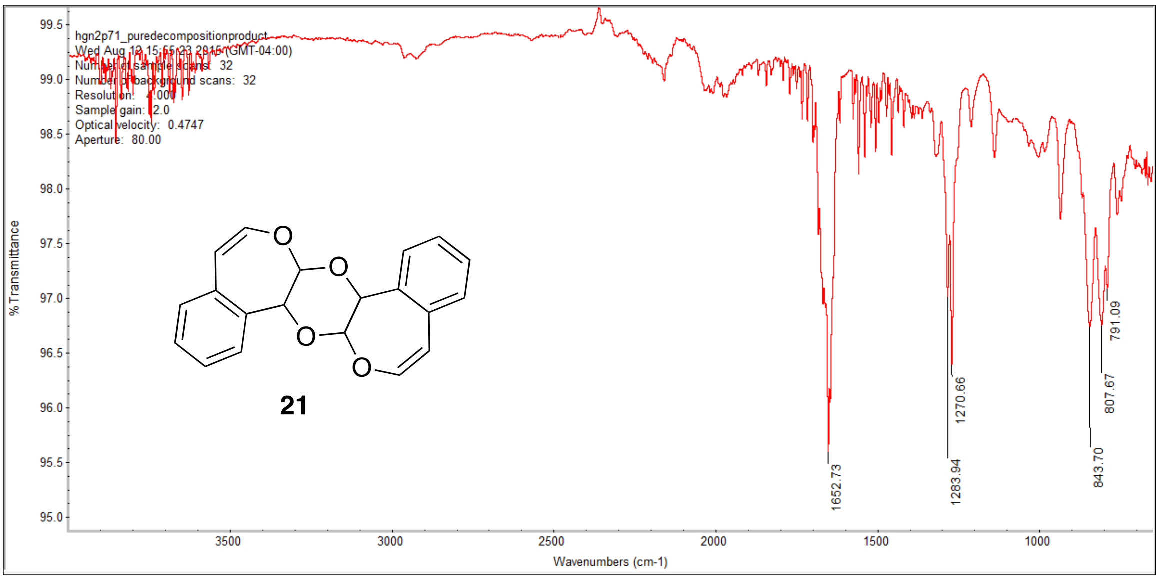

Figure S34. IR (neat) spectrum of **21**.

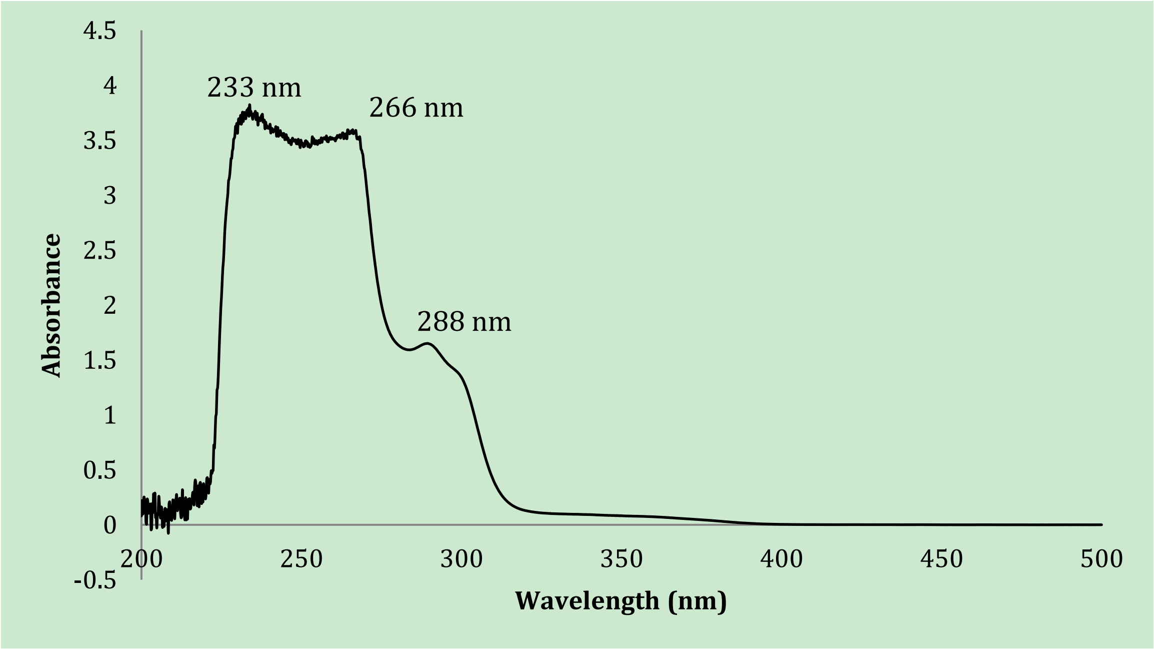

Figure S35. UV-Vis (0.2 mg/mL in dichloromethane) of **21**.

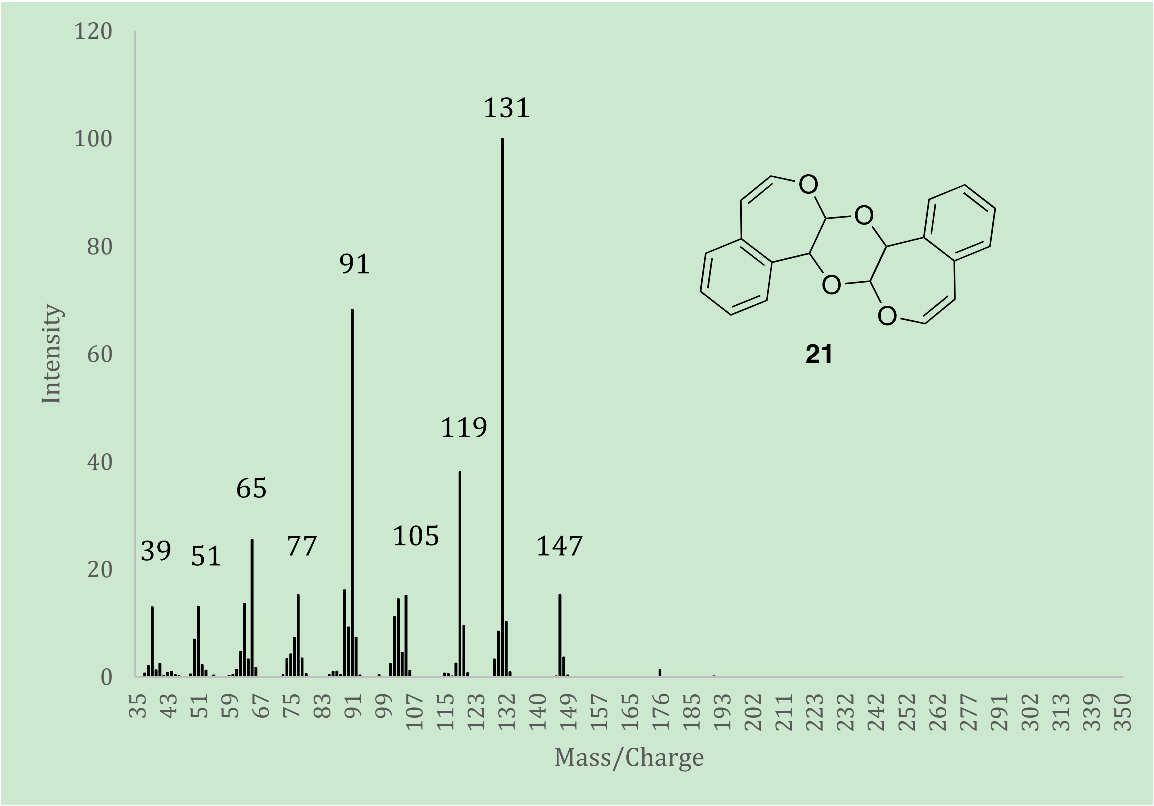

Figure S36. EI-MS of 21.

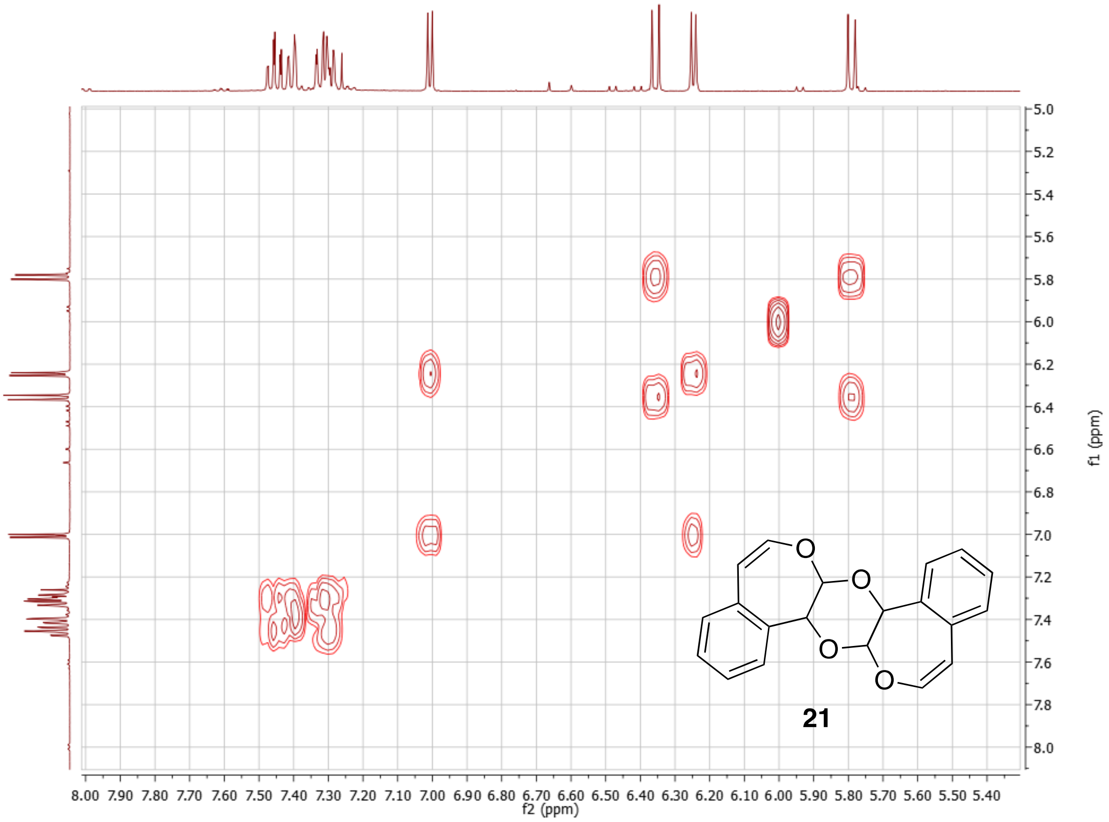

Figure S37. COSY (500 MHz, $CDCl_3$) of **21**.

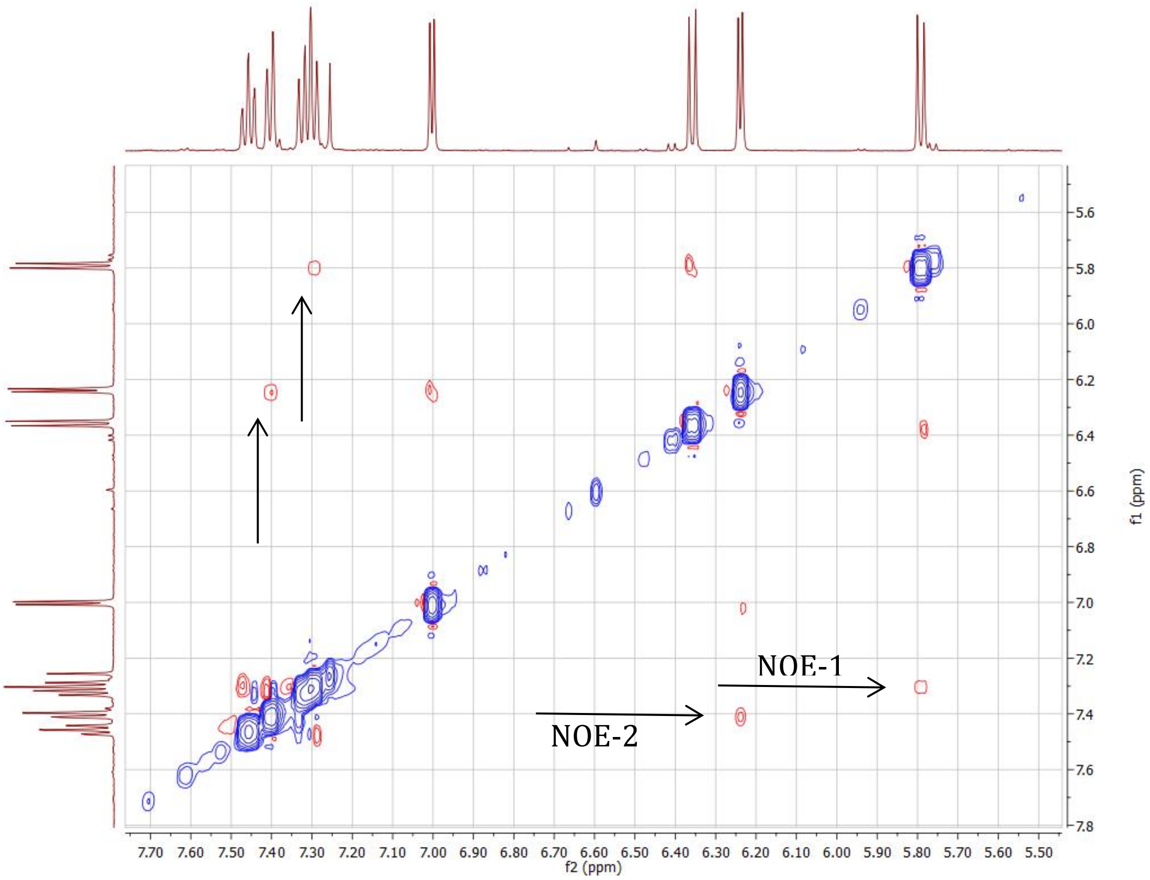

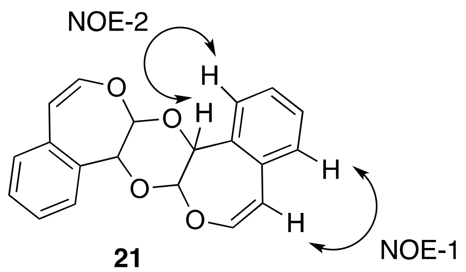

21

Figure S38. NOESY (500 MHz, $CDCl_3$) of **21**.

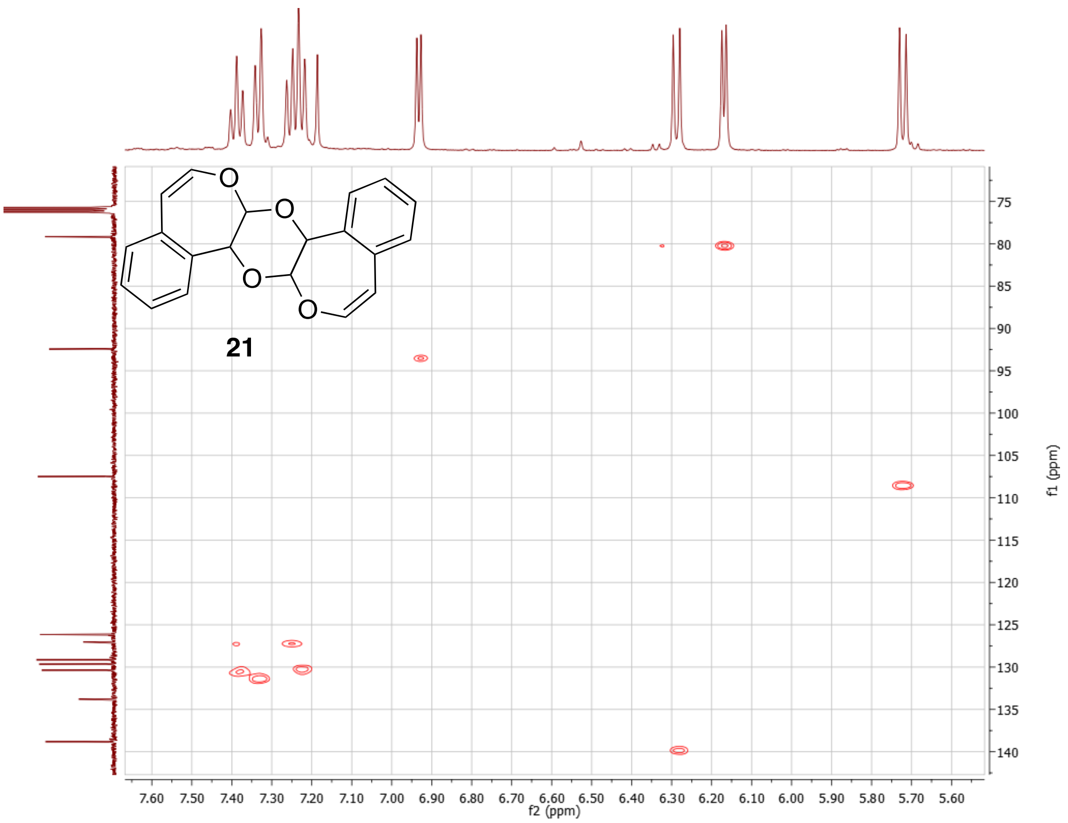

Figure S39. HMQC (500 MHz, $CDCl_3$) of **21**.

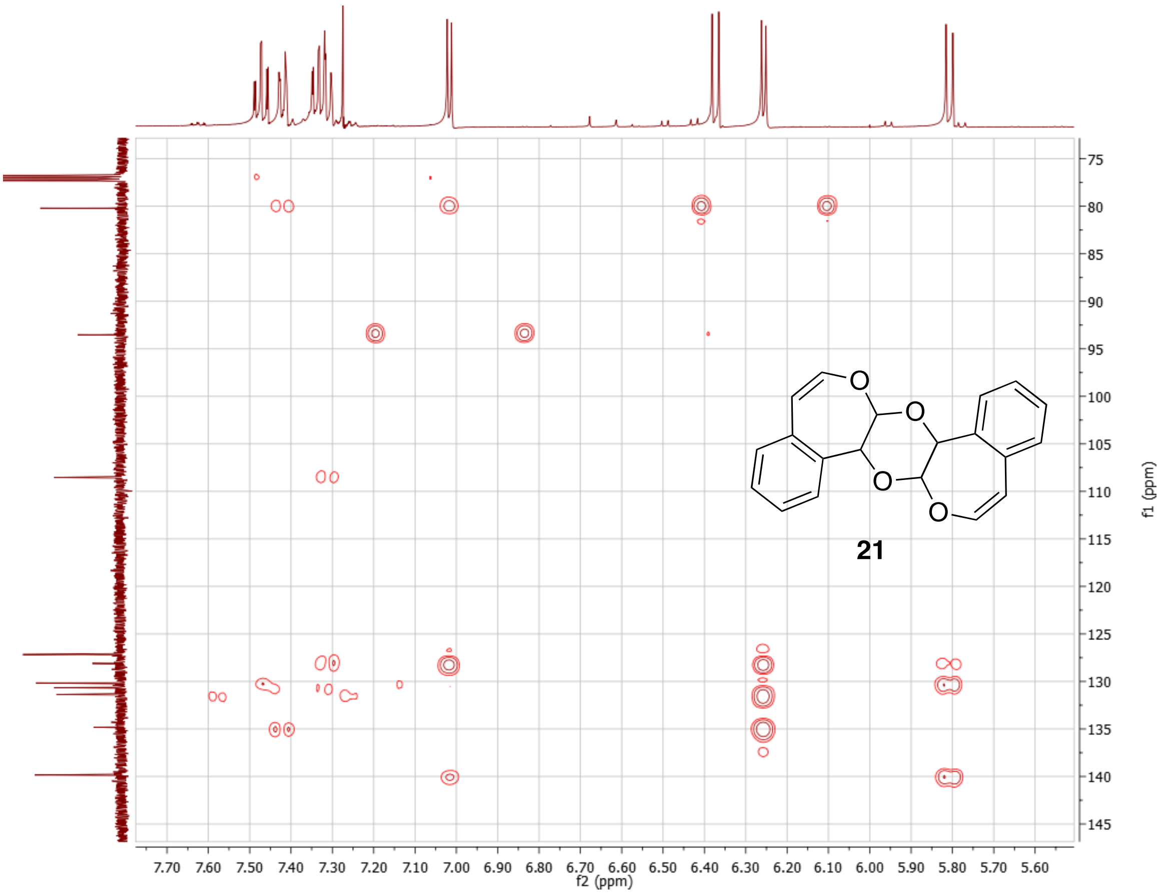

Figure S40. HMBC (500 MHz, $CDCl_3$) of **21** (J = 5 Hz).

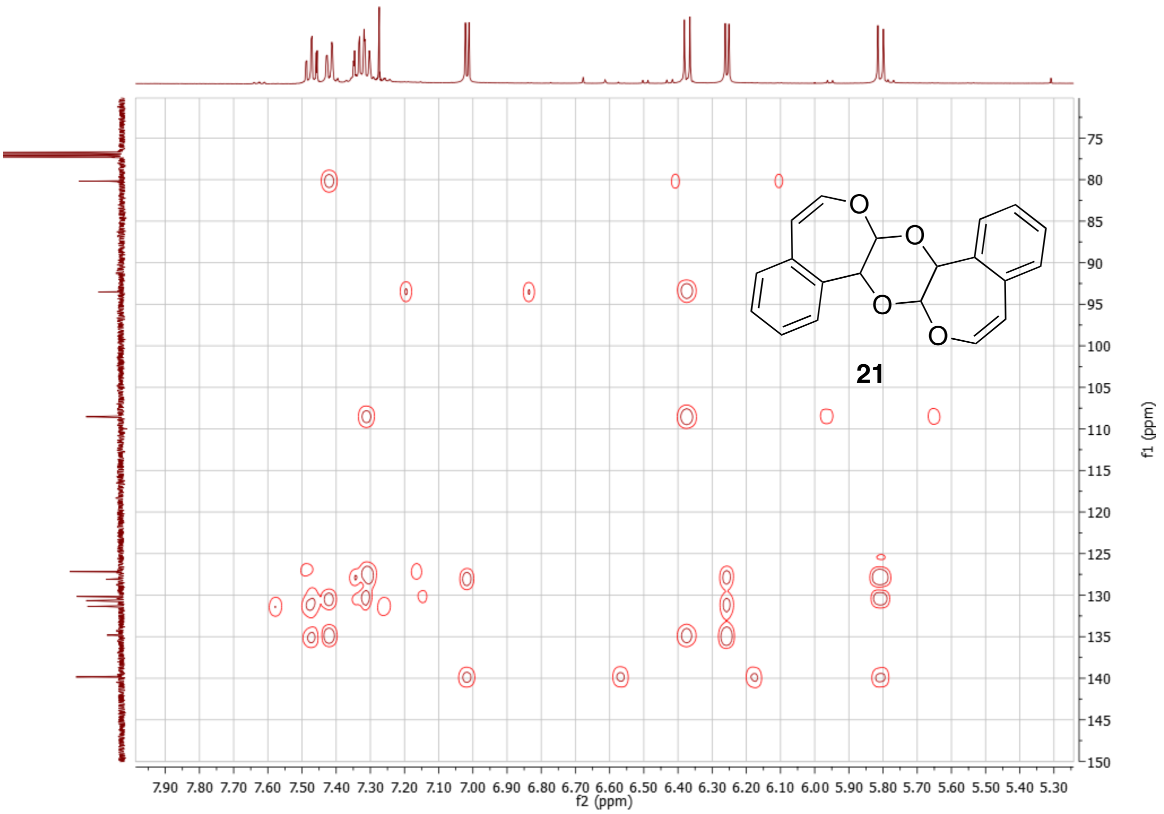

Figure S41. HMBC (500 MHz, CDCl3) of **21** (J = 8 Hz).

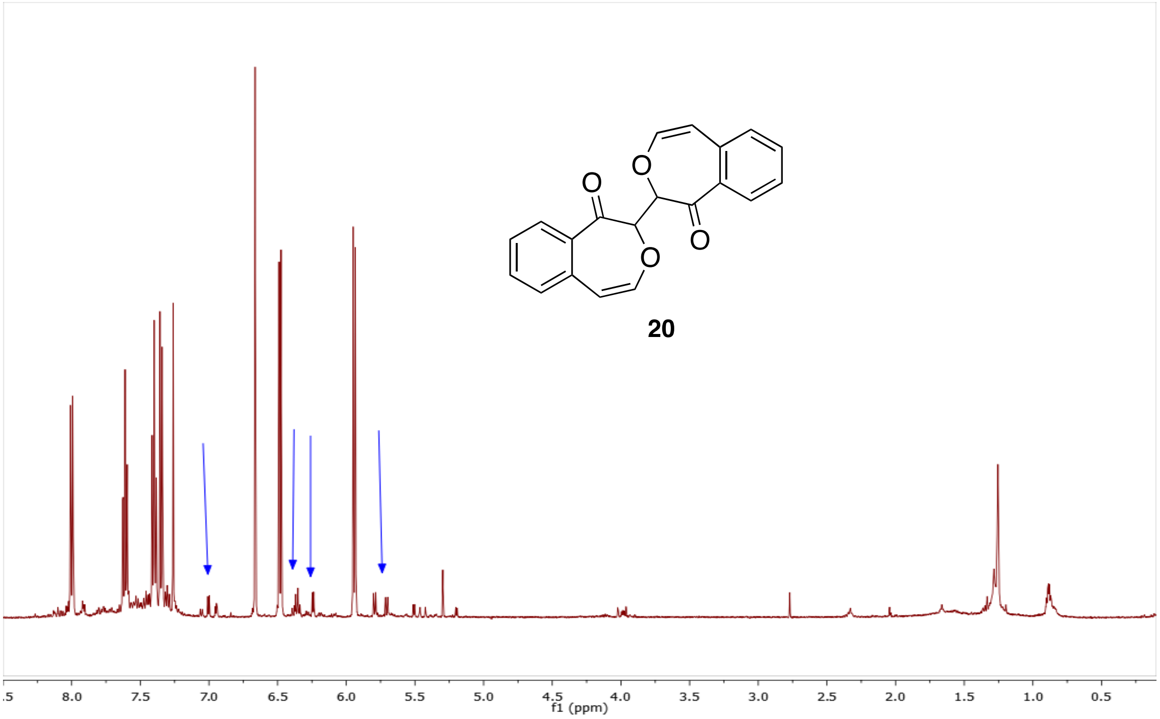

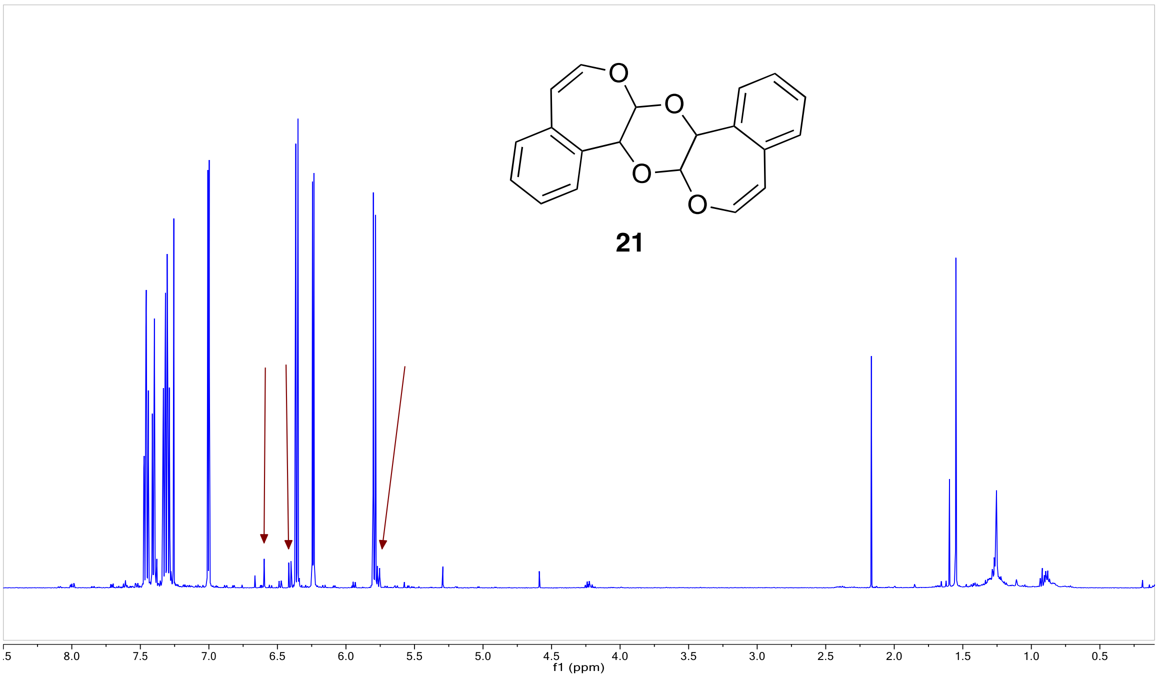

Figure S42. Top: **20** shows trace amount **21**. Bottom: **21** shows trace amount of **20**.

3. Calculations

R,R-**20** *meso*-**20**

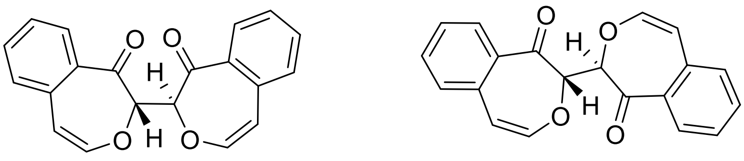

relative energy: 0 kcal/mol 1.42 kcal/mol

Figure S43. DFT calculations (B3LYP/6-31G**) of RR and meso isomers of **20**, respectively.

trans-**21** *cis*-**21**

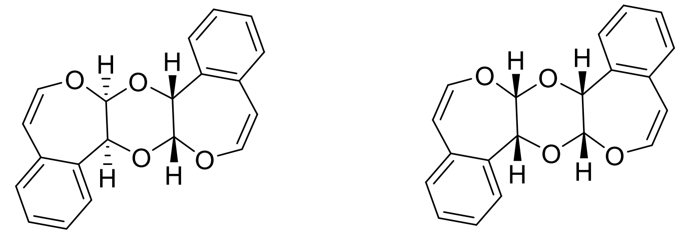

relative energy: 0 kcal/mol 5.69 kcal/mol

Figure S44. DFT calculations (B3LYP/6-31G**) of trans and cis isomers of **21**, respectively.

4. References

[1] Rabideau, P.W.; Burkholder, E.G. Metal-ammonia reduction and reductive alkylation of polycyclic aromatic compounds: nature of the anionic intermediates. *J. Org. Chem.* **1978**, *43*, 4283–4288.

[2] Paquette, L.A.; Barrett, J.H. 2,7-Dimethyloxepin - Organic Syntheses. *Org. Synth*. **1969**, *49*, 62.
